# Identification of membrane curvature sensing motifs essential for VPS37A phagophore recruitment and autophagosome closure

**DOI:** 10.1038/s42003-024-06026-7

**Published:** 2024-03-15

**Authors:** Yansheng Ye, Xinwen Liang, Guifang Wang, Maria C. Bewley, Kouta Hamamoto, Xiaoming Liu, John M. Flanagan, Hong-Gang Wang, Yoshinori Takahashi, Fang Tian

**Affiliations:** 1 Department of Biochemistry and Molecular Biology, The Pennsylvania State University, Hershey, PA 17033 USA; 2 Department of Pediatrics, Division of Pediatric Hematology and Oncology, Pennsylvania State University College of Medicine, Hershey, PA 17033 USA

**Keywords:** Biophysics, Biochemistry, NMR spectroscopy

## Abstract

VPS37A, an ESCRT-I complex component, is required for recruiting a subset of ESCRT proteins to the phagophore for autophagosome closure. However, the mechanism by which VPS37A is targeted to the phagophore remains obscure. Here, we demonstrate that the VPS37A N-terminal domain exhibits selective interactions with highly curved membranes, mediated by two membrane-interacting motifs within the disordered regions surrounding its Ubiquitin E2 variant-like (UEVL) domain. Site-directed mutations of residues in these motifs disrupt ESCRT-I localization to the phagophore and result in defective phagophore closure and compromised autophagic flux in vivo, highlighting their essential role during autophagy. In conjunction with the UEVL domain, we postulate that these motifs guide a functional assembly of the ESCRT machinery at the highly curved tip of the phagophore for autophagosome closure. These results advance the notion that the distinctive membrane architecture of the cup-shaped phagophore spatially regulates autophagosome biogenesis.

## Abstract (web summary)

Two membrane curvature-sensitive motifs in unstructured regions of the N-terminal domain of VPS37A are required for its phagophore recruitment and autophagosome closure.

## Introduction

During the process of macroautophagy, hereafter referred to as autophagy, cytoplasmic cargoes, including misfolded proteins and dysfunctional organelles, are sequestered into a double-membrane vesicle (autophagosome) prior to lysosomal delivery and subsequent degradation. The biogenesis of a functional autophagosome involves a specific and extensive sequence of membrane remodeling events, including nucleation, expansion, and sealing of the phagophore, a vesicle precursor^1–3^. While many of these steps are coordinated by autophagy-related (Atg) proteins^4^, we have recently demonstrated that specific components of the endosomal sorting complexes required for transport (ESCRT) machinery, such as VPS37A and CHMP2A, are required for sealing the cup-shaped phagophore and forming a double-membrane autophagosome^5–7^. Subsequent reports from other labs have corroborated the necessity of ESCRT components in phagophore closure both in budding yeast and in the context of mitophagy^8,9^. Together, these studies have established the critical role of ESCRT proteins in mediating phagophore closure during the biogenesis of autophagosomes.

The ESCRT machinery is an assembly of protein subcomplexes (ESCRT-I, -III) that function with the AAA ATPase VPS4 to draw opposing membranes together and mediate the final membrane scission reaction^10–12^. In the canonical ESCRT pathway, ESCRT-I and -II recognize upstream recruiting factors and mediate ESCRT-III targeting. Once recruited, ESCRT-III assembles into membrane-bound, filament-like structures that are subsequently rearranged and disassembled by VPS4 at the expense of ATP hydrolysis, leading to membrane constriction and abscission. In addition to autophagy, ESCRTs have been implicated in a variety of cellular membrane remodeling processes, including multivesicular body (MVB) formation, cytokinesis, plasma membrane and lysosome repair, nuclear pore reformation, exosome biogenesis, and virus budding^13,14^.

VPS37A, a subunit of the ESCRT-I complex, translocates to the phagophore during autophagy and directs ESCRT assembly for autophagosome closure^6,7^. The human VPS37A protein has 397 amino acids and consists of two domains (Supplementary Fig. 1a, b). Its N-terminal domain, spanning approximately the first 220 residues, is unique among its four mammalian homologs (VPS37A-D, Supplementary Fig. 1b). This region is predicted to encompass a Ubiquitin E2 variant-like (UEVL) domain (residues 24 to 125, previously referred to as a putative Ubiquitin E2 variant domain^6^), along with extensive stretches of unstructured regions that are poorly characterized. Deletion of the first 90 residues of VPS37A disrupts its localization to the phagophore^6^, suggesting that this region serves a specific function, although the exact nature of this process remains unclear. On the other hand, the C-terminal (~180 residues) of VPS37A, similar to its mammalian homologs (Supplementary Fig. 1b), contains a Mod(r) domain that is part of the core structure of the heterotetrameric ESCRT-I complex consisting of TSG101-VPS28-VPS37A-UBAP1. In a schematic model we built based on previous studies^15,16^ (Supplementary Fig. 1c), the N-terminal Ubiquitin E2 variant (UEV) domain of TSG101 and the C-terminal solenoid of overlapping Ubiquitin-associated (SOUBA) domains of UBAP1 are flexibly tethered to the core to recognize ubiquitinated cargos, and the C-terminal domain (CTD) of VPS28 is used to recruit ESCRT-II. The VPS37A N-terminal domain is presumably flexibly tethered to the core of ESCRT-I, but its function is unclear.

This study demonstrates that the VPS37A N-terminal domain selectively interacts with highly curved membranes in vitro and identifies two functionally important hydrophobic membrane-binding motifs in its unstructured regions. Mutations in these motifs result in impaired VPS37A localization to the phagophore, phagophore closure, and autophagy flux in vivo. We postulate that the preference for curved membranes exhibited by its N-terminal domain potentially guides (or contributes to guiding) VPS37A to the highly curved membrane edge of the phagophore. In addition, we show that despite the structural similarity between the core structure of the VPS37A UEVL domain and that of the TSG101 UEV domain, they are not functionally interchangeable. This suggests that the VPS37A UEVL domain harbors unique, albeit uncharacterized, functions related to autophagosome closure.

## Results

### VPS37A N-terminal domain selectively interacts with highly curved membranes

**Fig. 1:**
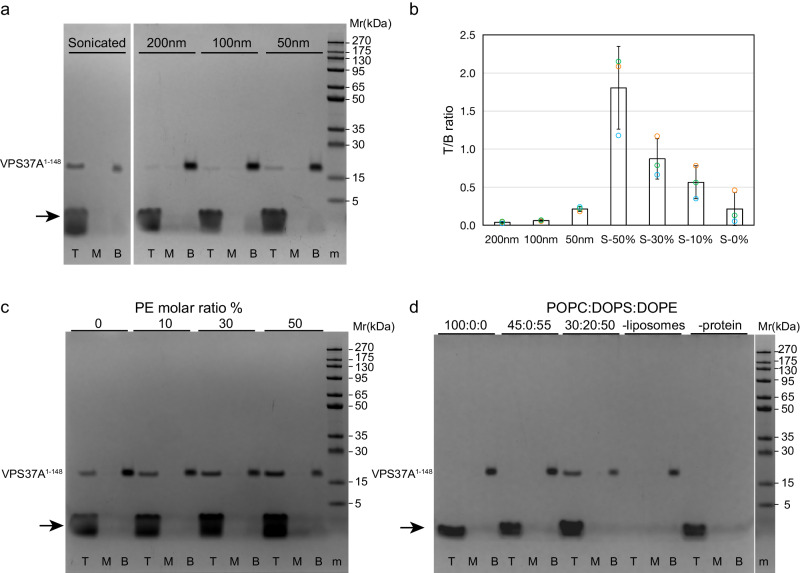
VPS37A^1–148^ selectively interacts with highly curved membranes. **a** Gel images of liposome flotation assays for VPS37A^1–148^ mixed with sonicated or extruded liposomes with membrane pore sizes of 50, 100, or 200 nm (POPC:DOPG:DOPE = 3:2:5, protein:lipid = 1:400, molar ratio). T, M, and B represent the top, middle, and bottom layers after centrifugation. The protein marker is indicated by m. The arrow indicates the lipid band. The amount of VPS37A^1–148^ in the top layer relative to that in the bottom layer (T/B) is quantitated by ImageJ and plotted in **b**. **b** Plots of VPS37A^1–148^ in top layer versus bottom layer (T/B) for extruded liposomes (POPC:DOPG:DOPE = 3:2:5) with membrane pore sizes of 50, 100, or 200 nm, and sonicated liposomes containing 0, 10, 30, or 50% PE (molar ratio, referred to as S-0%, S-10%, S-30%, and S-50%, respectively). Data are presented as mean ± SD (standard deviation). Quantifications were obtained from three separate measurements (*n* = 3). **c** Gel images of liposome flotation assays for VPS37A^1–148^ mixed with sonicated liposomes consisting of 20% DOPG and 0, 10, 30, or 50% of PE (molar ratio). Their plots are shown in **b**. The protein marker is indicated by m. The arrow indicates the lipid band. **d** Gel images of liposome flotation assays for VPS37A^1–148^ mixed with sonicated liposomes containing 0 or 55% of DOPE (molar ratio) without negatively charged lipids, and with sonicated liposomes (POPC:DOPS:DOPE = 3:2:5, protein:lipid = 1:400, molar ratio). Samples with VPS37A^1–148^ proteins alone (-liposomes) or liposomes alone (-protein) were performed as controls. The protein marker is indicated by m. The arrow indicates the lipid band.

Yeast Vps37 (yVps37) binds membranes with acidic lipids in vitro via its N-terminal basic helix^17^, and multiple autophagy-related proteins, such as Atg1, Atg3, Atg13, Atg14L, and Atg16L1^18^, preferentially interact with highly curved membranes. These observations led us to investigate whether the human VPS37A N-terminal domain interacts with the membrane. Based on a previous biological study^19^, we used a construct consisting of residues 1 to 148 (VPS37A^1–148^) that is predicted to contain a putative UEVL domain (Supplementary Fig. 1a). In a liposome flotation assay as shown in Fig. 1a, b and Supplementary Fig. 2a, about ~65% of the VPS37A^1–148^ protein was detected in the top (T) lipid-containing fraction with sonicated liposomes (20.0 nm average radius measured by dynamic light scattering, DLS; POPC:DOPG:DOPE = 3:2:5, molar ratio). However, with three different preparations of larger liposomes (average radii of 38.5, 48.9, and 69.3 nm determined by DLS and prepared by extrusion with 50, 100, and 200 nm filters, respectively), the protein was predominantly detected in the aqueous solution fraction (bottom, B), indicating that the protein minimally binds to larger and less curved liposomes. In these experiments, the liposome composition was similar to that previously employed in our in vitro LC3-PE (phosphatidylethanolamine) liposome conjugation assay^20,21^ (since DOPS is a potential reaction substrate, DOPG instead of DOPS was used as negatively charged lipids). We also tested liposomes prepared with DOPS lipids. As shown in Fig. 1a, b and Supplementary Fig. 2b, c, DOPG-containing and DOPS-containing liposomes produced consistent results. Thus, we conclude that VPS37A^1–148^ interacts with model membranes and its interaction depends on vesicle size; VPS37A^1–148^ clearly shows a preference for small lipid vesicles with highly curved membranes.

Hydrophobic insertion is one of the primary molecular mechanisms for membrane curvature recognition^22,23^. Packing defects in the outer leaflet of the lipid bilayer are a hallmark of highly curved membranes, and curvature-sensitive molecules preferentially embed into surfaces that display this feature. By extension, introducing packing defects into the bilayer by incorporating lipids with small headgroups, such as PEs, should strengthen the membrane binding of curvature-sensitive molecules^24^. To determine how VPS37A^1-148^ targets small, highly curved membranes, we repeated the liposome flotation assay with sonicated liposomes containing different percentages of PE lipids. As shown in Fig. 1b, c and Supplementary Fig. 2b, c, the amount of VPS37A^1–148^ in the top fraction positively correlates with increasing percentages of PE lipids in liposomes. This observation suggests that the membrane-binding potential of VPS37A^1–148^ is enhanced by PE lipids, consistent with the concept that bilayer packing defects contribute to its membrane binding. In addition, VPS37A^1–148^ does not float with liposomes without DOPG or DOPS lipids (Fig. 1d), indicating that its membrane binding requires negatively charged lipids. These results are consistent with a model in which VPS37A^1–148^ selects curved membranes through hydrophobic insertion, with both hydrophobic and electrostatic interactions stabilizing its binding to the membrane.

### VPS37A N-terminal domain has two membrane-binding motifs

We employed high-resolution NMR spectroscopy to identify the sequence(s) responsible for the observed membrane curvature-sensitive interaction of VPS37A^1–148^. All resonances from non-proline residues except Ser24, His89, and N-terminal 13 residues (BMRB: 51558) were assigned in an aqueous solution (Supplementary Fig. 3). Since there are only four unassigned peaks in the 2D ^1^H-^15^N correlation spectrum, resonances from the majority of the first 13 residues were not observed, likely due to exchange broadenings. In addition, analyses of secondary chemical shifts of ^13^C_α_ and ^13^C_β_ indicate that residues 14 to 20 and residues 132 to 148 are unstructured, consistent with the AlphaFold prediction (Supplementary Fig. 1a).

**Fig. 2:**
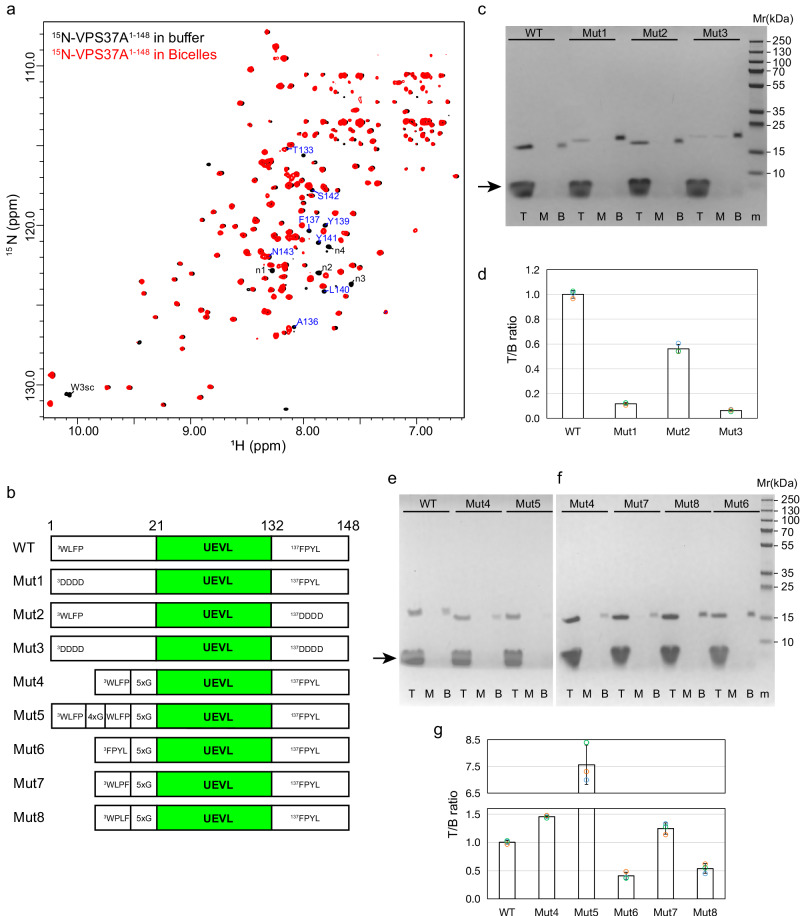
Two conserved hydrophobic motifs in VPS37A^1–148^ interact with the membrane. **a** Overlay of ^15^N-labeled VPS37A^1–148^ TROSY spectra in the absence (black) and presence (red) of bicelles (DMPC:DMPG:DHPC = 4:1:10, molar ratio, *q* = 0.5). Several perturbed resonances are labeled with their assignments. Unassigned resonances are labeled with n1 to n4. **b** Diagrams of VPS37A^1–148^ constructs for in vitro study. **c** Gel images of liposome flotation assays for VPS37A^1–148^ wildtype (WT), Mut1, Mut2, and Mut3 mixed with sonicated liposomes (POPC:DOPG:DOPE = 3:2:5, protein:lipid = 1:400, molar ratio). T, M, and B represent the top, middle, and bottom layers after centrifugation. The protein marker is indicated by m. The arrow indicates the lipid band. **d** Plots of VPS37A^1–148^ WT, Mut1, Mut2, and Mut3 in the top layer relative to the bottom layer in flotation experiments (**c**) using sonicated liposomes (POPC:DOPG:DOPE = 3:2:5). The T/B ratios of mutants are normalized to the ratio of VPS37A^1–148^ WT using the same batch of sonicated liposomes. The amount of protein in the top layer relative to the bottom layer (T/B) is quantitated by ImageJ. Data are presented as mean ± SD from three independent experiments (*n* = 3 for each construct). **e** Gel images of liposome flotation assays for VPS37A^1–148^ WT, Mut4, and Mut5 mixed with sonicated liposomes (POPC:DOPG:DOPE = 3:2:5, protein:lipid = 1:400, molar ratio). T, M, and B represent the top, middle, and bottom layers after centrifugation. The arrow indicates the lipid band. **f** Gel images of liposome flotation assays for Mut4, Mut5, Mut6, Mut7, and Mut8 mixed with sonicated liposomes (POPC:DOPG:DOPE = 3:2:5, protein:lipid = 1:400, molar ratio). T, M, and B represent the top, middle, and bottom layers after centrifugation. Protein marker is indicated by m. **g** Plots of VPS37A^1–148^ WT, Mut4, Mut5, Mut6, Mut7, and Mut8 in the top relative to the bottom layer (T/B) in flotation experiments (**e**, **f**) using sonicated liposomes (POPC:DOPG:DOPE = 3:2:5). The T/B ratios of mutants are normalized to the ratio of VPS37A^1–148^ WT using the same batch of sonicated liposomes. The amount of protein in the top layer relative to the bottom layer (T/B) is quantitated by ImageJ. Data are presented as mean ± SD from three independent experiments (*n* = 3 for each construct).

Bicelles have recently been shown to be an effective model for highly curved membranes because the dynamic planar surfaces of bicelles are loosely packed and presumably mimic the type of packing defects found in highly curved membranes^20^. Figure 2a shows an overlay of 2D ^1^H-^15^N correlation spectra of VPS37A^1–148^ in the presence and absence of bicelles (DMPC:DMPG:DHPC = 4:1:10, *q* = 0.5). These spectra were acquired at 15 °C to increase the lifetime of VPS37A^1–148^ NMR resonances in bicelles, which generally disappear within an hour at 25 °C. In the presence of bicelles, several peaks were shifted, including resonances from residues Ala136 to Asn143, the indole amide proton of residue Trp3, and four unassigned resonances (which are from 13 unassigned residues of the N-terminal disordered region as described above). Based on sequence alignment (Supplementary Fig. 4), these perturbed residues cluster around two highly conserved regions that contain bulky hydrophobic amino acids, ^3^WLFP and ^137^FPYL (highlighted in yellow), which straddle the predicted UEVL domain. These two regions are particularly interesting because inserting hydrophobic moieties into one leaflet of lipid bilayers is a common mechanism for membrane curvature recognition and generation.

To examine the relative contributions of ^3^WLFP and ^137^FPYL motifs to membrane binding, we prepared three mutants of the VPS37A^1–148^ construct: Mut1, where ^3^WLFP is mutated to ^3^DDDD; Mut2, where ^137^FPYL is mutated to ^137^DDDD; and Mut3, where both ^3^WLFP and ^137^FPYL are mutated to DDDD (Fig. 2b). Figure 2c, d show the results of liposome flotation experiments for these variants. Compared with VPS37A^1–148^ wildtype (WT) protein, the ratios of membrane-bound to free Mut1, Mut2, and Mut3 were reduced by ~90%, ~45%, and ~94%, respectively. Consistent results were observed for these three mutants with DOPS-containing liposomes (Supplementary Fig. 2d, e). These observations suggest that both hydrophobic motifs contribute to membrane interactions, but the loss of hydrophobic interactions involving the ^3^WLFP motif has a larger effect.

The N-terminal ^3^WLFP motif is connected to the UEVL domain by a flexible linker of 13 residues, including one basic and seven polar residues. We examined the contributions of the linker to the membrane binding of VPS37A^1–148^ by replacing the linker with 5 Gly residues (Mut4, Fig. 2b). As shown in Fig. 2e, g, the ratio of membrane-bound to free Mut4 increased by ~50% relative to the VPS37A^1–148^ WT protein. The enhancement in membrane binding is presumably due to a reduction in the hydrophilic nature of the linker and/or its increased flexibility. Additionally, we prepared a construct containing two WLFP motifs separated by a linker of 4 Gly residues (Mut5, Fig. 2b). Mut5 showed dramatically enhanced membrane binding, with the ratio of membrane-bound to free Mut5 increased by ~650% relative to the VPS37A^1–148^ WT protein and by ~400% relative to the Mut4 (Fig. 2e, g). Conversely, replacing the ^3^WLFP with the ^137^FPYL motif (Mut6) resulted in a ~73% reduction in the ratio of membrane-bound to free Mut6 relative to Mut4 (Fig. 2f, g), suggesting that the ^3^WLFP motif has a higher affinity for membranes than the ^137^FPYL motif. This is consistent with the results described above for Mut1 and Mut2 (Fig. 2c, d and Supplementary Fig. 2d, e).

We further investigated whether the structure of the ^3^WLFP motif affects its membrane-binding potential by creating a set of mutants where the relative positions of ^4^Leu, ^5^Phe, and ^6^Pro residues were switched (^3^WLFP was mutated to ^3^WLPF, Mut7; ^3^WLFP was mutated to ^3^WPLF, Mut8; Fig. 2b). Compared with VPS37A^1–148^ Mut4 proteins, the ratios of membrane-bound to free Mut7 and Mut8 were decreased by ~15% and ~63% (Fig. 2f, g), respectively. These results are consistent with our previous observations that Pro often plays a critical structural role in membrane curvature recognition^20,25^.

### Membrane-binding motifs in the VPS37A N-terminal domain mediate phagophore targeting of ESCRT-I for autophagosome closure

**Fig. 3:**
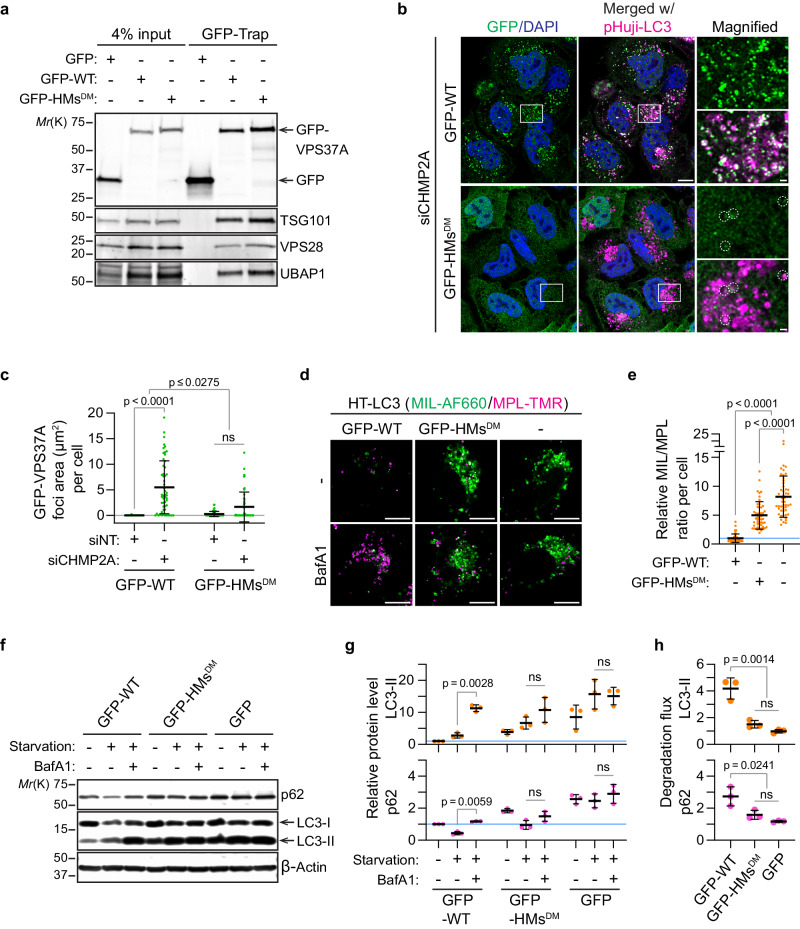
Mutations in the VPS37A N-terminal hydrophobic membrane-binding motifs impair phagophore targeting of the ESCRT-I complex for autophagosome closure. **a**–**h** VPS37A KO U-2 OS cells were stably transduced with GFP-tagged VPS37A wildtype (GFP-WT) or a mutant with both ^3^WLFP and ^137^FPYL motifs mutated to DDDD (GFP-HMs^DM^). In **b**, **d** the resultant cells were further transduced with pHuji-LC3 and HaloTag (HT)-LC3, respectively. **a** Immunoblot analysis of whole-cell lysates (input) and immunoprecipitates (GFP-Trap) from the indicated cells. **b** Confocal images of cells that were transfected with the indicated siRNAs for 45 h and starved for 3 h. Magnified images in the boxed areas are shown in the right panels. GFP-HMs^DM^ signals detected on LC3-positive structures are indicated by dotted circles. Scale bars represent 10 μm, and 1 μm in the magnified images. **c** Quantification of GFP-VPS37A-positive foci area per cell in **b** (*n* ≥ 50) with two-way ANOVA followed by Tukey’s multiple comparisons. **d** Confocal images of cells that were starved for 3 h in the presence or absence of Bafilomycin A1 (BafA1) and subjected to the HT-LC3 autophagosome completion assay using the indicated membrane-impermeable (MIL) and -permeable (MPL) HaloTag ligands. Scale bars represent 10 μm. **e** Quantification of the MIL/MPL fluorescence intensity ratio for each cell in the starvation plus BafA1 treatment group (*n* = 50) with one-way ANOVA followed by Tukey’s multiple comparisons. The data shown are relative to the mean of GFP-WT-expressing cells. **f** Immunoblot analysis of cells that were starved for 3 h in the presence or absence of BafA1. p62 is a cargo degraded by autophagy, and LC3-II (a covalent conjugation form of LC3 to the amino headgroup of phosphatidylethanolamine lipid) is an autophagic marker. **g**, **h** Dot plots of LC3-II and p62 band intensities relative to the mean of untreated GFP-WT-expressing cells (**g**) and LC3-II and p62 degradation ratios relative to GFP-WT-expressing cells (**h**) in **f** (*n *= 3). Data in **g** and **h** are analyzed with two-way and one-way ANOVA followed by Tukey’s multiple comparisons, respectively. All values in the graphs are mean ± SD. ns, not significant.

To assess the role of hydrophobic membrane-binding motifs (HMs), namely ^3^WLFP and ^137^FPYL, in targeting the ESCRT-I complex to the phagophore, the VPS37A wildtype and a mutant with both the ^3^WLFP and ^137^FPYL motifs mutated to DDDD (HMs^DM^) were transduced into VPS37A knockout (KO) U-2 OS human osteosarcoma cells^6^. Each construct was fused to a green fluorescent protein (GFP) or mScarlet fluorescent protein for subcellular localization monitoring. Consistent with our previous study^6^, GFP-tagged wildtype VPS37A (GFP-WT) successfully formed a complex with other ESCRT-I components, including TSG101, VPS28, and UBAP1 (Fig. 3a) and accumulated on LC3-labeled phagophores upon depletion of the ESCRT-III component CHMP2A (Fig. 3b, c). Depletion of CHMP2A serves to stabilize the ESCRT assembly, enabling us to visualize the VSP37A complex on the phagophore^6^. In contrast, most of the GFP-HMs^DM^ signals exhibited diffuse fluorescence throughout the cytoplasm, with only a few associating with LC3-positive structures, even though this mutant is successfully integrated into the ESCRT-I complex (Fig. 3a–c). Moreover, unlike the mScarlet-tagged VPS37A WT, the mScarlet-HMs^DM^ mutant did not co-accumulate with another ESCRT-I component, GFP-VPS28, on LC3-positive structures in VPS37A KO cells after CHMP2A depletion (Supplementary Fig. 5). These results collectively demonstrate that HMs are required for targeting the ESCRT-I complex to the phagophore.

To explore the functional significance of the VPS37A HMs in ESCRT-mediated autophagosome closure, we performed the HaloTag-LC3 autophagosome completion assay (HT-LC3 assay)^5^. In this assay, we employed the Alexa Fluor 660-conjugated membrane-impermeable HaloTag ligands (MIL) and tetramethylrhodamine (TMR)-conjugated membrane-permeable HaloTag ligands (MPL) to selectively label open and closed autophagosomal structures: MIL^+^MPL^-^ for phagophores and MPL^+^ for closed autophagosomes. As autophagosome-enclosed HT-LC3 proteins are subjected to lysosomal degradation^5^, we conducted the assay with and without the lysosomal inhibitor Bafilomycin A1 (BafA1). In nutrient-starved VPS37A KO cells, MPL^+^ closed autophagosomes did not accumulate upon BafA1 treatment, while MIL^+^MPL^-^ structures (phagophores) increased both in the presence and absence of BafA1 (Fig. 3d), suggesting a deficiency in autophagosome closure. Importantly, this impaired autophagosome closure was only rescued by the expression of GFP-WT, not GFP-HMs^DM^, as demonstrated by a decrease of MIL^+^MPL^-^ puncta under starvation conditions and an increase of MPL^+^ puncta under starvation conditions in the presence of BafA1 (Fig. 3d). The MIL/MPL ratio in GFP-HMs^DM^-expressing VPS37A KO cells was slightly lower than that in VPS37A KO cells (Fig. 3e). This can be attributed to the residual presence of GFP-HMs^DM^ on LC3-positive structures (Fig. 3b), suggesting limited activity in autophagosome closure. In line with this finding, as shown in Fig. 3f–h, GFP-HMs^DM^ proved ineffective in reestablishing autophagic flux in VPS37A KO cells. This was determined by assessing the BafA1-sensitive lysosomal degradation of the membrane-bound form of LC3 (LC3-II) and the autophagic receptor p62. These results suggest that the curvature-sensitive membrane interaction mediated by HMs in the VPS37A N-terminal domain plays an essential role in autophagosome closure and subsequent cargo degradation. Notably, mutations in either the ^3^WLFP or ^137^FPYL motifs did not affect the ability of GFP-VPS37A to restore autophagic flux in VPS37A KO cells (Supplementary Fig. 6), implying functional redundancy between these motifs. However, in the liposome flotation assay described above (Fig. 2c, d), the ^3^WLFP motif, when compared to ^137^FPYL, exerts a larger influence on the membrane binding of VPS37A^1–148^. One possible explanation for this discrepancy is that the ^137^FPYL motif in the full-length VPS37A might exhibit increased membrane affinity in a cellular environment.

### The VPS37A UEVL domain is essential for the proper assembly of the ESCRT machinery required for autophagosome closure

**Fig. 4:**
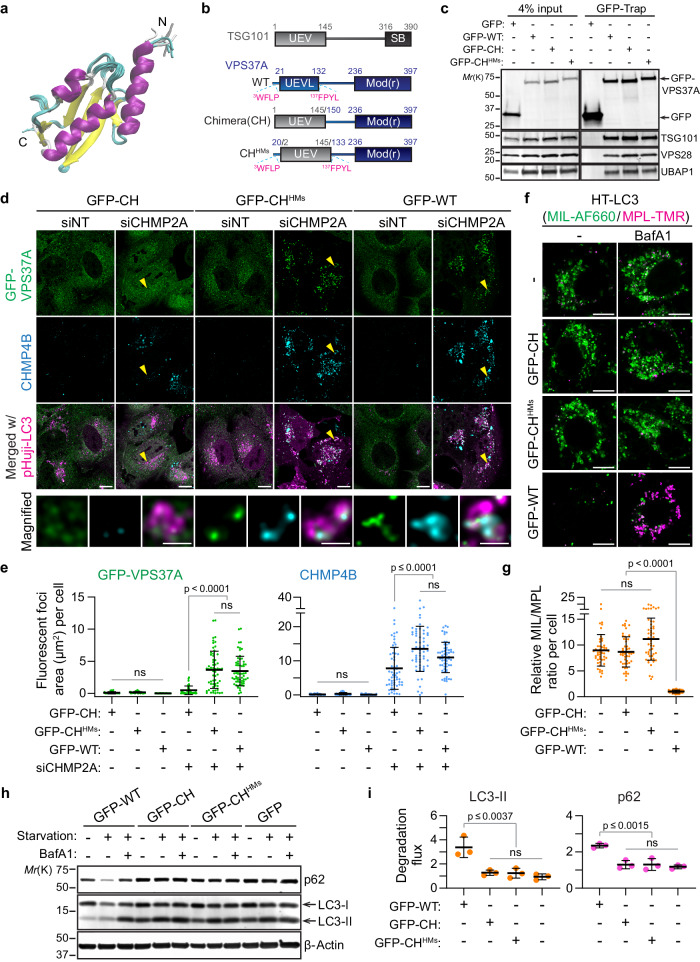
The VPS37A UEVL domain is indispensable for autophagosome closure. **c**–**i** VPS37A KO U-2 OS cells were stably transduced with the indicated GFP-tagged VPS37A constructs. **a** Overlay of ten lowest-energy NMR structures of VPS37A^21–148^. The unstructured region of residues 133 to 148 is not shown. N and C indicate N- and C- terminals, respectively. **b** Diagram of VPS37A and mutants for in vivo study. **c** Immunoblot analysis of whole-cell lysates (input) and immunoprecipitates (GFP-Trap) from the indicated cells. **d** Confocal images of cells that were transfected with the indicated siRNAs for 45 h and starved for 3 h. Magnified images in the arrowhead-indicated areas are shown in the bottom panels. Scale bars represent 10 μm, and 1 μm in the magnified images. **e** Quantification of GFP-VPS37A-positive and CHMP4B-positive foci area per cell in **d** (*n* ≥ 60) with one-way ANOVA followed by Tukey’s multiple comparisons. **f** Confocal images of cells that were transduced with HT-LC3, starved for 3 h in the presence or absence of BafA1, and subjected to the HT-LC3 autophagosome completion assay using the indicated HaloTag ligands. Scale bars represent 10 μm. **g** Quantification of the MIL/MPL fluorescence intensity ratio for each cell in the starvation plus BafA1 treatment group (*n* = 50) with the Kruskal–Wallis test followed by Dunn’s multiple comparisons. The data shown are relative to the mean of GFP-WT-expressing cells. **h** Immunoblot analysis of cells that were starved for 3 h in the presence or absence of BafA1. **i** Dot plots of LC3-II and p62 degradation ratios relative to GFP-WT-expressing cells in **h** (*n* = 3) with one-way ANOVA followed by Tukey’s multiple comparisons. All values in the graphs are mean ± SD. ns, not significant.

The predicted UEVL domain in the VPS37A N-terminus does not have notable sequence homology with any proteins of known structure. To determine its structure, we employed high-resolution NMR spectroscopy. Since the first 20 residues of VPS37A^1–148^ are unstructured in solution (as described above), we prepared a construct comprising residues 21 to 148 of VPS37A (VPS37A^21–148^) to facilitate its structure determination. The 2D ^1^H-^15^N correlation spectrum of VPS37A^21–148^ closely overlaps with that of the initial VPS37A^1–148^ constructs, as shown in Supplementary Fig. 7. Minor shifts in these two spectra indicate that removing the first 20 residues does not perturb the structural integrity of the construct. The solution structure of VPS37A^21–148^ determined with a combination of NOEs, RDCs, and torsion angle restraints is shown in Fig. 4a (PDB ID: 8E22, BMRB ID:31039, Table 1). As expected, residues 21 to 131 adopt the characteristic α/β fold commonly found in canonical UEV domains, while residues 132 to 148 are unstructured. The reported C_α_ RMSDs between the NMR-derived and the AlphaFold predicted structures of VPS37A^21–131^ by Chimera are ~1.0 Å (Supplementary Fig. 8a).

**Table 1:** NMR and refinement statistics for VPS37A^21–148^

	Protein
**NMR distance and dihedral constraints**	
Distance constraints	
Total NOE	2385
Intra-residue	538
Inter-residue	1847
Sequential (|i – j | = 1)	666
Medium-range (1 < |i – j | ≤ 4)	417
Long-range (|i – j | ≥ 5)	764
Intermolecular	0
Hydrogen bonds	0
Total dihedral angle restraints	
*ϕ*	97
*ψ*	97
Total RDC restraints	
Phage	74
**Structure statistics**	
Violations (mean and s.d.)	
Distance constraints (Å)	0.066 ± 0.001
Dihedral angle constraints (°)	0.667 ± 0.093
Max. dihedral angle violation (°)	7.7
Max. distance constraint violation (Å)	0.67
Deviations from idealized geometry	
Bond lengths (Å)	0.004 ± 0.000
Bond angles (°)	0.611 ± 0.006
Impropers (°)	0.504 ± 0.010
Q factor^a^	0.12 ± 0.01
Average pairwise r.m.s. deviation^b^ (Å)	
Heavy	0.9
Backbone	0.3

^a^Definition of the RDC Q factor is given in the literature^43^.

^b^Pairwise r.m.s. deviation was calculated among 10 refined structures. Evaluated for secondary structure elements: 24–39, 44–45, 51–58, 61–68, 79–83, 86–87, 101–104, 112–125, 129–130.

The UEV fold is a characteristic feature of a protein family that is structurally homologous to the canonical E2 Ubiquitin-conjugating enzymes but lacks the catalytic cysteine residue^26^. Given the structural similarity of the VPS37A UEVL domain to other UEV proteins (Supplementary Fig. 8b), we examined whether the VPS37A UEVL domain is functionally interchangeable with TSG101 UEV. To explore this, we first constructed a GFP-tagged chimeric construct, denoted as GFP-CH, where residues 1 to 149 of VPS37A were replaced with the corresponding sequence (residues 1 to 145) of TSG101 (Fig. 4b). Despite the successful formation of the ESCRT-I complex, GFP-CH failed to localize to the phagophore for the recruitment of the downstream ESCRT-III subunit CHMP4B (Fig. 4c, d). This observation is consistent with our above findings that HMs are required for VPS37A’s localization to the phagophore and the subsequent steps of phagophore closure (Fig. 3b, c). We next examined whether flanking the TSG101 UEV domain with HMs enabled the targeting of ESCRTs to the phagophore. We prepared another chimeric construct, denoted as GFP-CH^HMs^ (Fig. 4b), where residues 21 to 132 of VPS37A were replaced with residues 1 to 145 of TSG101. It is worth noting that, in vitro, a TSG101 UEV chimeric mutant with the first 20 residues of VPS37A (containing the ^3^WLFP motif) enhances the binding of the TSG101 UEV domain to liposomes (Supplementary Fig. 9). We found that cells stably transduced with GFP-CH^HMs^ accumulated both GFP-CH^HMs^ and CHMP4B on LC3-positive structures to a similar extent as GFP-WT-expressing cells after CHMP2A depletion (Fig. 4d, e). These results indicate that GFP-CH^HMs^ can rescue the defect in ESCRT targeting observed in VPS37A KO cells.

To examine whether the successful targeting of GFP-CH^HMs^ to the phagophore can lead to functional ESCRT assembly for autophagosome closure, we performed the HT-LC3 assay. As shown in Fig. 4f, g, the expression of GFP-CH^HMs^ failed to rescue the defect in the formation of an MPL^+^ sealed autophagosome in VPS37A KO cells and resulted in the accumulation of MIL^+^MPL^-^ structures upon induction of autophagy by nutrient-starvation. Additionally, the autophagic assay demonstrated impaired lysosomal degradations of LC3-II and p62 in these cells (Fig. 4h, i). These observations are similar to the outcomes seen in cells that express GFP-CH and GFP-HMs^DM^ mutants, both of which are unable to target VPS37A to the phagophore as described above (Figs. 4f–i and 3d–h). Together, our data indicate that, in VPS37A KO cells that express GFP-CH^HMs^, ESCRT assemblies are dysfunctional despite the restoration of phagophore localization, underscoring the critical yet enigmatic role of the VPS37A UEVL domain in autophagosome sealing.

## Discussion

Autophagosome formation constitutes a multifaceted and dynamically evolving process that involves membrane remodeling. We have recently discovered that several components of the ESCRT machinery, including the human proteins VPS37A, VPS28, TSG101, CHMP2A, and VPS4, are necessary for the sealing of the cup-shaped phagophore and the subsequent formation of the autophagosome^5,6^. Importantly, VPS37A recognizes the phagophore and recruits a subset of ESCRT components to complete phagophore closure. Here we report that, in vitro, the VPS37A N-terminal domain, unique among its four mammalian homologs, selectively interacts with highly curved membranes via two hydrophobic motifs in its disordered regions. The functional relevance of this interaction is demonstrated by in vivo mutagenesis studies; mutations in these two motifs disrupt its phagophore localization and impair phagophore closure and autophagic flux.

**Fig. 5:**
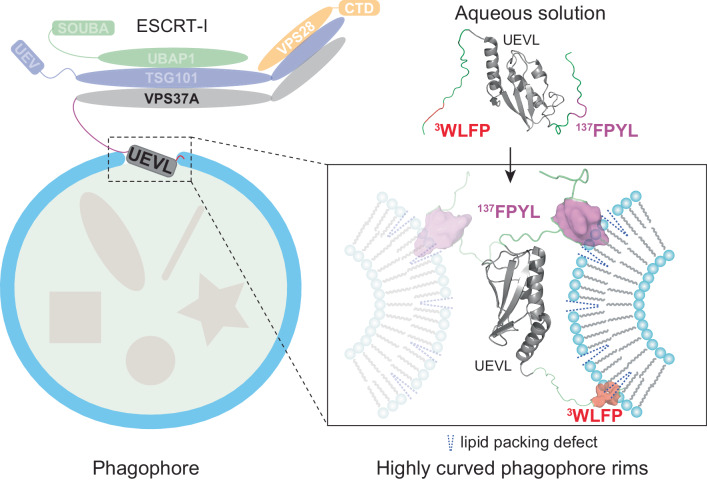
A model for the recruitment of VPS37A to the phagophore. Interactions of highly curved membrane edges of the phagophore with two hydrophobic motifs of the VPS37A N-terminal domain recruit (or contribute to the recruitment of) the ESCRT-I complex to the phagophore.

Membrane geometry has been recognized as an essential component of the microenvironments in which membrane fusion and fission, protein localization, trafficking, and signaling occur. Membrane curvature can regulate the activity and determine the subcellular localization of some proteins. For instance, human ArfGAP1 and Atg3 proteins display enhanced enzymatic activity when interacting with small highly curved lipid vesicles but show little to no activity on large and less curved lipid vesicles^24,27^. Bacterial DivIVA and SpoVM proteins recognize membranes with negative or slight positive curvatures for their intracellular localizations, respectively^28–31^. These proteins insert an amphipathic helix or a hydrophobic loop into one leaflet of the bilayer to sense membrane curvature, which is one of the most common curvature recognition mechanisms^20,21,32^. Membrane curve-sensitive binding is a recurring theme for autophagy-related proteins, including Atg1, Atg3, Atg13, Atg14L, and Atg16L1^18^. The selective binding of Atg3 to membranes with high curvatures is implicated in targeting the protein to the tip of the phagophore and regulating its activity during phagophore expansion. Here we identify two hydrophobic motifs, ^3^WLFP and ^137^FPYL, that mediate the preferential interaction of VPS37A^1–148^ with small and highly curved liposomes. Both sequences contain bulky hydrophobic sidechains and are effective for membrane insertion. Substituting them with Asp residues markedly reduces VPS37A^1–148^ binding to liposomes in vitro and impairs phagophore targeting of ESCRT-I in vivo. Therefore, even though additional interactions may be necessary, we propose that the highly curved leading edges of the phagophore function as a geometric cue, and their interactions with the two evolutionarily conserved hydrophobic motifs of VPS37A play a pivotal role in recruiting or contributing to the recruitment of ESCRT-I to the phagophore (Fig. 5). During the revision of this manuscript, the Deretic group reported the involvement of LC3A and GABARAP, two ATG8 family proteins, in VPS37A recruitment via a putative LIR motif for autophagosome sealing^33^. Notably, the presumed VPS37A region interacting with LC3A and GABARAP includes the ^3^WLFP motif identified in this study. While these findings align with previous data showing that the disruption of lipidation in ATG8 family proteins leads to the accumulation of unclosed autophagosomal membranes^34,35^, we found that the ^3^WLFP/^3^DDDD mutation of VPS37A had little effect on autophagic flux (Supplementary Fig. 6). Furthermore, minimal chemical shift perturbations were detected in ^1^H-^15^N correlation spectra of ^15^N- labeled VPS37A^1–148^ in the presence or absence of unlabeled LC3A (Supplementary Fig. 10a, b), as well as in ^1^H-^15^N correlation spectra of ^15^N-labeled LC3A in the presence or absence of unlabeled VPS37A^1–148^ (Supplementary Fig. 10c, d). These data indicate that VPS37A^1–148^ does not interact with LC3A in vitro.

The initiation of ESCRT assembly involves recognizing site-specific elements, ubiquitinated proteins, and membranes by ESCRT-I^11–13^. A heterotetrameric ESCRT-I complex, composed of TSG101, VPS28, one of the VPS37 homologs, and one of the UMA proteins (UBAP1 or MVB12A/B), contains at least two targeting modules flexibly linked to the long-stretched core structure: TSG101 UEV for site-specific elements or ubiquitinated proteins^36,37^, and either UBAP1 SOUBA for ubiquitinated proteins^15^ or MVB12-associated β-prism (MABP) for membranes^38^. This study has identified two critical motifs in the VPS37A N-terminal domain, namely ^3^WLFP and ^137^FPYL, as curvature-sensitive membrane-binding modules of ESCRT-I. These motifs are essential for targeting ESCRT to the phagophore during autophagy without affecting the formation of the ESCRT-I complex itself. Notably, while UBAP1, a major UMA protein selectively incorporated into the VPS37A-containing ESCRT-I complex, is crucial for Ubiquitin-dependent EGFR sorting in the MVB pathway^16^, its absence has a negligible effect on autophagosome closure^6^. In contrast, deletion of the VPS37A N-terminus impairs autophagosome closure but not EGFR sorting^6^. These results suggest the utilization of different modules for ESCRT-I recruitment: Ubiquitin-binding for receptor sorting and membrane-binding for autophagosome closure. We postulate that distinct membrane geometries at ESCRT-I recruitment sites may be exploited for selective targeting in ESCRT-dependent membrane remodeling processes. The membrane curvature-selective interaction of the VPS37A N-terminal domain revealed in this study provides a model for how the highly curved rim of phagophores recruits the ESCRT-I complex. By comparison, in the MVB pathway, the ESCRT-I complex is recruited to nearly “flat” endosomal membranes during initiation via interactions with ESCRT-0 and ubiquitinated cargos.

VPS37A KO cells that express GFP-CH^HMs^ restored ESCRT recruitment to the phagophore and appeared to be capable of assembling and disassembling ESCRT-III. However, this complex remained defective for autophagosome closure (Fig. 4). The non-functional ESCRT complex observed in GFP-CH^HMs^-expressing cells cannot simply be explained by complex destabilization on the membranes because the level of ESCRT-I accumulation on LC3-positive structures induced by CHMP2A depletion in these cells was comparable to that in wildtype VPS37A-expressing cells. This situation differs from cells that express the ESCRT-I scaffold formation-defective VPS28 mutant in which phagophore accumulation of ESCRT-I is destabilized^7^. These observations support an essential yet undocumented function of the VPS37A UEVL domain during phagophore closure that is distinct from the TSG101 UEV domain. Consistently, NMR chemical shift perturbation experiments showed that, unlike TSG101 UEV, the VPS37A UEVL domain does not bind to Ubiquitin as it lacks the Ubiquitin interacting region of TSG101 UEV (Supplementary Fig. 11). Thus, factors (including lipids) that interact with the VPS37A UEVL remain to be defined. As shown in Supplementary Fig. 12, while few chemical shift perturbations were observed in the ^1^H-^15^N correlation spectra of a ^15^N-labeled VPS37A^1-148^ variant with ^3^WLFP/^3^DDDD and ^137^FPYL/^137^DDDD mutations (Mut3) in a freshly prepared bicelle sample, multiple resonances shifted after this sample stayed inside the magnet at 15 °C for ~8 h. While detailed characterizations of these perturbations and their functional relevance are beyond the scope of the current study, future investigations are warranted to determine how the VPS37A UEVL domain, in conjunction with the two hydrophobic membrane-binding motifs, orchestrates the spatiotemporal process of autophagosome closure.

## Methods

### Reagents

The following antibodies were used for immunoblotting (IB) and immunofluorescence (IF): β-ACTIN (IB, Sigma-Aldrich, A5441, 1:10,000); CHMP4B (IF, ABclonal, A7402, 1:100); GFP (IB, Cell Signaling, 2956, 1:1,000); MAP1LC3B (IB, Novus, NB100-2220, 1:3,000; IF, MBL, M152-3, 1:200); and p62 (American Research Products, 03-GP62-C, 1:4,000). ON-TARGETplus SMART Pool Non-targeting (D-001810-10) and CHMP2A (L-020247-01) siRNAs were obtained from GE Healthcare Dharmacon. pCDH1-CMV-GFP-VPS37A WT-Puro was generated as described previously^6^ and used as a template to generate lentiviral GFP-VPS37A ^3^WLFP/^3^DDDD and ^137^FPYL/^137^DDDD (HMs^DM^)-expression vectors by Gibson Assembly using the primers listed in Supplementary Table 1. pCDH1-CMV-GFP-TSG101(N-terminus)-VPS37A (C-terminus) Chimera (GFP-CH)-Puro and pCDH1-CMV-GFP-CH with the VPS37A HMs (GFP-CH^HMs^)-Puro was constructed by Gibson Assembly using pCDH1-CMV-GFP-VPS37A WT-Puro, Human TSG101 cDNA from Sanford Simon through Addgene (116925)^39^ and the primers listed in Supplementary Table 1. All other reagents were obtained from the following sources: bafilomycin A1 (LC Laboratories, B-1080); 4’,6-diamidino-2-phenylindole, dihydrochloride (DAPI) (BD Bioscience, 564907); digitonin (Sigma-Aldrich, D141); membrane-impermeable HaloTag Ligand (MIL) (Promega, Alexa Fluor 660-conjugated, G8471); membrane-permeable HaloTag Ligand (MPL) (Promega, tetramethylrhodamine-conjugated, G8251); normal goat serum (Sigma-Aldrich, G9023); paraformaldehyde (Electron Microscopy Sciences, 15710); Triton X-100 (Sigma-Aldrich, T8532); XF Plasma Membrane Permeabilizer (XF-PMP) (Seahorse Bioscience, 102504-100).

### Cell culture, viral transduction, and autophagy induction

U-2 OS and 293T/17 (CRL-11268) cells obtained from American Type Culture Collection were maintained in McCoy’s 5A Medium supplemented with 10% fetal bovine serum (FBS) or Dulbecco’s Modification of Eagle’s Medium (DMEM) supplemented with 10% FBS, respectively. VPS37A knockout U-2 OS cells were generated as described previously^6^. Lentiviral production and transduction were conducted using the Invitrogen ViraPower Lentiviral Expression System as described previously^6^. To induce autophagy, cells were rinsed three times with phosphate-buffered saline (PBS) and incubated with amino acid-free DMEM in the presence or absence of 100 nM BafA1 as described previously^6^.

### Immunoblotting and autophagic flux assay

Total cell lysates were prepared in radio-immunoprecipitation assay buffer (150 mM NaCl, 10 mM Tris-HCl, pH 7.4, 0.1% SDS, 1% Triton X-100, 1% Deoxycholate, 5 mM EDTA, pH 8.0) containing protease inhibitors and subjected to SDS-PAGE followed by immunoblotting with indicated antibodies as described previously^5^. The signal intensities of LC3-II and p62 were quantified using Image Studio version 5 software (LI-COR Biotechnology) and normalized to the respective value of β-actin. Autophagic degradation rates were calculated according to the guidelines^40^. The formulas to calculate each event were: normalized values of LC3-II and p62 in cells starved with BafA1 over those in cells starved without BafA1.

### Co-immunoprecipitation

Total cell lysates prepared in 0.5% NP-40 lysis buffer (10 mM Tris/Cl, pH 7.5, 150 mM NaCl, 0.5 mM EDTA, 0.5% NP-40) containing protease inhibitors were subjected to immunoprecipitation with GFP-Trap beads (Chromotek, gtma). The resulting immunoprecipitates were washed three times with lysis buffer and subjected to immunoblotting.

### Immunofluorescence labeling, HaloTag-LC3 autophagosome completion assay, and confocal microscopy

Cells were seeded on Lab-Tek II Chambered Coverglass, Chamber Slide (Nunc, 154941). For immunofluorescence, cells were fixed in 4% paraformaldehyde-PBS for 10 min, permeabilized with 100 µg/mL digitonin (LC3B) or 0.25% Triton X-100 (CHMP4B) for 5 min, blocked in 10% normal goat serum for 1 h, and incubated with primary antibodies overnight at 4 °C followed by secondary antibodies for 1 h at room temperature (RT). Cells were then incubated with 1 µg/mL DAPI at room temperature for 10 min to visualize nuclei. A HaloTag-LC3 autophagosome completion assay was performed as described previously^5^. Briefly, cells were incubated in 1× MAS buffer (220 mM mannitol, 70 mM sucrose, 10 mM KH_2_PO4, 5 mM MgCl_2_, 2 mM HEPES, and 1 mM EGTA) containing 3 nM XF-PMP and 3.5 µM MIL-AF660 at 37 °C for 15 min, fixed in 4% PFA for 5 min, and incubated with 5 µM TMR for 30 min at RT. All fluorescence images were obtained at RT using a Leica AOBS SP8 laser-scanning confocal microscope (63× oil-immersion [1.2 numerical aperture lens] or 63× water [1.4 numerical aperture lens]) with the highly sensitive HyD detectors and the Leica image acquisition software LAX. The images were deconvolved using Huygens deconvolution software (Scientific Volume Imaging), and analyzed using Imaris software (Bitplane), Fiji software (ImageJ), and ilastik^41^ software without gamma adjustment.

### Statistics and reproducibility

Numerical source data for all graphs are provided in the Supplementary Data. Statistical analysis was performed using a one-way or two-way ANOVA test followed by Tukey’s multiple comparisons test or Kruskal–Wallis test followed by Dunn’s multiple comparisons in Graph Pad Prism 7.0. The threshold for statistical significance for each test was set at 95% confidence (*p* < 0.05). All data were performed at least *n* = 3 independent experiments.

### Protein expression and purification

DNA sequences encoding residues 1 to 148 of VPS37A (VPS37A^1-148^) and residues 1 to 145 of the TSG101 UEV domain were separately subcloned into the pET28a expression vector at the BamHI/XhoI site with a His6-tag, a T7-tag and a thrombin cleavage site between the T7-tag and N-termini. LC3A with a His6-tag at the N-terminus subcloned into the pET28a expression vector was kindly provided by Dr. Xuejun Jiang (Memorial Sloan Kettering Cancer Center, New York). All VPS37A^1-148^ and TSG101 EUV mutants were generated using the Q5 Site-Directed Mutagenesis Kit (New England Biolabs). The corresponding primers are listed in Supplementary Table 1, and all constructs were verified by sequencing. Plasmids were then transformed into chemically competent Rosetta™(DE3) pLysS cells for expression. Typically, a single colony was selected to grow in a small volume of LB medium overnight at 37 °C as a starter culture and then inoculated into a large volume of LB medium (for unlabeled proteins) or M9 medium supplemented with D-glucose (or 3 g/L D-glucose-^13^C_6_, Cambridge isotope laboratories, CLM-1396) and ^15^NH_4_Cl (1 g/L, Cambridge isotope laboratories, NLM-467; or Sigma-Aldrich, 299251) for ^15^N (or ^15^N/^13^C) labeled samples at 37 °C. After OD_600_ reached 0.6 to 0.8, the temperature was lowered to 25 °C, and cells were induced with 0.5 mM IPTG for ~16 h. Cells were harvested by centrifugation, and the pellets were stored at −80 °C until use.

Cell pellets for VPS37A^1-148^ or its mutants were homogenized with a lysis buffer of 20 mM phosphate, pH 7.5, 300 mM NaCl, 2 mM β-mercaptoethanol (BME), complete protease inhibitor cocktail (Roche, 11836170001), and 0.1% triton X-100 (Alfa Aesar, A16046) and were lysed by sonication on ice with 2 s on and 7 s off intervals for 18 min total duration or by microfluidics. Cell debris was removed by centrifugation (Sorvall RC5B Plus Refrigerated Centrifuge) at 26,900 × *g* at 10 °C for 30 min. Supernatants were collected and loaded onto a Ni-NTA column (HisTrap HP, Cytiva 17524801). The column was washed with PBS buffers of 20 mM phosphate, pH 7.5, 300 mM NaCl, 2 mM BME without and with 20 mM imidazole and then eluted with a PBS buffer containing 500 mM imidazole. The elution of VPS37A^1-148^ or its mutants from the Ni-NTA column was concentrated and exchanged into a buffer containing 50 mM HEPES, pH 7.5, 150 mM NaCl, and 2 mM BME, followed by the addition of 0.1% (v/v) TWEEN20 (Fisher Biotech, BP337-500) and 50 units thrombin (Sigma-Aldrich, 605157) to remove the T7-tag and His6-tag for an overnight agitation at 4 °C. The solution was then subjected to a Ni-NTA column. The flow-through was collected and further purified by size-exclusion chromatography using an S200 column (HiLoad 16/60 Superdex 200, Cytiva 28-9893-35) with a running buffer of 50 mM HEPES, pH 7.5, 1 M NaCl, and 1 mM DTT. Purified VPS37A^1-148^ or mutant proteins were exchanged into a buffer of 20 mM PBS, pH 6.5 (or 50 mM HEPES, pH 6.5 to 7.5), 150 mM NaCl. TSG101 UEV and its mutant were purified with a similar protocol. LC3A was purified through a Ni-NTA column and then an S200 column with corresponding buffers as described above. The purified protein was exchanged into a buffer of 50 mM HEPES, pH 6.8 (or 7.0 for LC3A), 150 mM NaCl, and 2 mM TCEP. Protein concentration was assayed using a Nanodrop (ND-1000 Spectrophotometer, Thermo Scientific, 2353-30-0010).

### Liposome flotation assays

Liposomes (4 mM lipids) were prepared in double-distilled H_2_O (ddH_2_O) by sonication or extrusion with membrane pore sizes of 50, 100, and 200 nm^20^. Briefly, POPC (Avanti Polar Lipids, 850457), DOPG (Avanti Polar Lipids, 840475), DOPS (Avanti Polar Lipids, 840035), DOPE (Avanti Polar Lipids, 850725) in chloroform were added to a glass tube in corresponding molar ratio and dried to a thin film by spinning with heat for half an hour using a condenser rotor SpeedVac, followed by lyophilization overnight once the volatile organics were removed. Lipids were rehydrated with ddH_2_O for 1 h at 42 °C, with vortex, and then frozen at −80 °C every 15 min. For the preparation of sonicated liposomes, the rehydrated lipids were then for bath sonication (BRANSON 3510R-MT Bransonic Ultrasonic Cleaner). The lipids were sonicated for 4× 15-min intervals until clear. For extruded liposomes, the rehydrated lipids were passed through a 0.05, 0.1, or 0.2 μm filter 11 times (SPARTAN HPLC Syringe Filter) in a liposome extruder (Avanti Polar Lipids) after the freeze-thaw steps. The extruded liposomes were collected and stored at room temperature. In the liposome flotation assays^24^, typically, 2 μM proteins were incubated with 800 μM liposomes in buffer A containing 50 mM HEPES, pH 7.5, and 150 mM NaCl (total volume of 300 μL) at room temperature for 1 h. The 300 μL mixtures were adjusted to a 40% OptiPrep Density gradient medium by mixing with 200 μL 100% (w/v) OptiPrep Density gradient medium (Sigma, D1556). The mixture was transferred to the bottom of a 3.5 mL, Open-Top Thickwall Polypropylene Tube (Beckman Coulter, 349622, 13 × 51 mm) mm at the bottom, followed by a 416.7 μL middle layer of 30% OptiPrep Density gradient medium and an 83.3 μL top layer of 0% OptiPrep Density gradient medium which were prepared separately in buffer A. Each tube was then subjected to centrifugation (Beckman, Optima XPN-80, A95765) at 200,000 × *g* in a sw55Ti rotor (Beckman, 34196) at 10 °C for 4 h. 125 μL samples from the top, middle, and bottom portions of the gradient were collected and 15 μL of each were mixed with 4 μL 4x loading buffer and heated for 3 min before SDS-PAGE gel electrophoresis (SurePAGE 10% Bis-Tris, GenScript, M00666; 150 V for 30 min).

### NMR spectroscopy and structure determination

Typical NMR samples consisted of 0.05 to 0.5 mM labeled proteins in a buffer of 20 mM PBS, pH 6.5 (or at specified buffer conditions) containing 150 mM NaCl and 0.02% NaN_3_. For residual dipolar coupling measurements, 140 μM ^15^N-labeled VPS37A^21-148^ was dissolved in 50 mM HEPES, pH 6.5, and 250 mM NaCl containing 6.5 mg/mL phage Pf1 (ASLA BIOTECH, P-50-P). For bicelle samples, 50 μM ^15^N-labeled VPS37A^1-148^ were mixed with a final concentration of 12% (w/v) bicelles (DMPC:DMPG:DHPC = 4:1:10, molar ratio, *q* = 0.5) in 25 mM HEPES, pH 7.0, 150 mM NaCl. Bicelles were prepared as previously described^20^. Typically, DMPC (Avanti Polar Lipids, 850345) and DMPG (Avanti Polar Lipids, 840445) were mixed with ddH_2_O, frozen at −80 °C, and thawed at 42 °C for 3 freeze-thaw cycles. DHPC (Avanti Polar Lipids, 850305) in ddH_2_O was then added to the mixture and quickly vortexed to form a clear solution. The solution was frozen at −80 °C and slowly thawed at room temperature for use. All NMR experiments were conducted at 25 °C except the one in bicelles that was collected at 15 °C (since the sample precipitates after NMR collection at 25 °C). All NMR data were acquired on a Bruker 600 MHz spectrometer except the NOESY data that were acquired on a Bruker 850 MHz spectrometer. Both instruments are equipped with cryoprobes. The data were processed using NMRPipe3.0 and analyzed using NMRViewJ (Version 9.1.0-b55 with Java 1.8.0). Backbone resonance assignments were carried out using triple resonance HNCO, HN(CA)CO, HNCA, HN(CO)CA, HNCACB, and HN(CO)CACB experiments (Bruker Topspin3.2 pulse sequence library). Aliphatic and aromatic sidechain resonance assignments were obtained from 3D ^13^C-edited HCCHTOCSY, ^13^C-edited NOESY, ^15^N-edited NOESY, and HBHA(CBCACO)NH, 2D Hb(CbCgCd)Hd, and Hb(CbCgCdCe)He spectra (Bruker Topspin3.2 pulse sequence library). The 3D ^13^C-NOESY, ^15^N-NOESY experiments (Bruker Topspin3.2 pulse sequence library) were collected with 150 ms mixing time. ^15^N-^1^H RDCs were measured using the IPAP-HSQC experiment^42^.

Torsion angle constraints were derived from C_α_, C_β_, N, and C’ chemical shifts using TALOS+. NOE assignments were obtained by a combined manual and automated analysis with CYANA 3.0. The structures were calculated with XPLOR-NIH 3.3. PSVS and PDB validation servers were used to analyze the ten lowest-energy structures.

### Reporting summary

Further information on research design is available in the Nature Portfolio Reporting Summary linked to this article.

### Supplementary information

Supplementary_information

Description of Additional Supplementary Files

Supplementary Data

Reporting summary

## Data Availability

Source images for representative western blots shown in Fig. 3a, f, Fig. 4c, h, and Supplementary Fig. 6 are provided in Supplementary Figs. 13–15 in Supplementary information. Numerical source data are provided in the Supplementary Data (an Excel file). NMR resonance assignments have been deposited with the BMRB with accession numbers 51558 for VPS37A^1-148^ and 31039 for VPS37A^21-148^. NMR structure has been deposited with the Protein Data Bank with accession ID 8E22. All other data supporting the study’s findings are available from the corresponding authors upon request.
