# Supplementary material for: Identification of membrane curvature sensing motifs essential for VPS37A phagophore recruitment and autophagosome closure
Source: Commun Biol. 2024 Mar 15;7:334. doi: 10.1038/s42003-024-06026-7 (PMC10942982; doi:10.1038/s42003-024-06026-7)
Supplement: Supplementary file 1 — Supplementary_information [file 42003_2024_6026_MOESM1_ESM.pdf]

Supplementary Information

Identification of Membrane Curvature Sensing Motifs Essential for VPS37A Phagophore Recruitment and Autophagosome Closure

Yansheng Ye,[1*§] Xinwen Liang,[2*] Guifang Wang, [1] Maria C Bewley, [1] Kouta Hamamoto,[2] Xiaoming Liu,[2] John M. Flanagan,[1] Hong-Gang Wang,[2] Yoshinori Takahashi[2§] and Fang Tian[1§]

[1] Department of Biochemistry and Molecular Biology, The Pennsylvania State University, Hershey, PA USA, 17033

[2] Department of Pediatrics, Division of Pediatric Hematology and Oncology, Pennsylvania State University College of Medicine, Hershey, PA USA, 17033

* These authors contributed equally to this work.

§ To whom correspondence should be addressed: Fang Tian (ftian@psu.edu), Yoshinori Takahashi (ytakahashi@pennstatehealth.psu.edu), or Yansheng Ye (yanshengye@pennstatehealth.psu.edu).

a

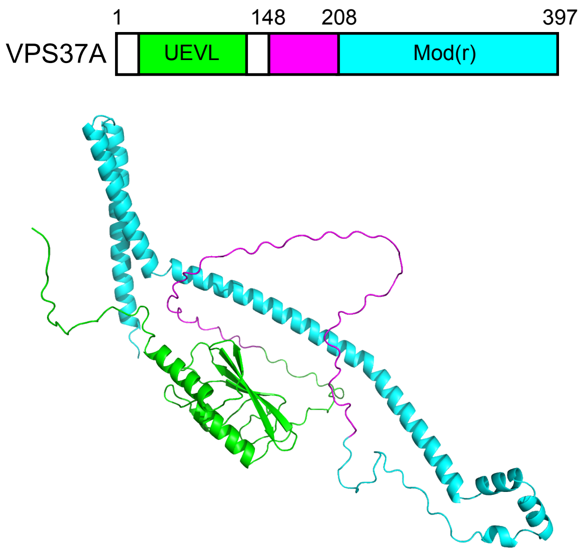

b

```
VPS37A MSWLFPLTKSASSSAAGSPGGLTSLQQQKQRLIESLRNSHSSIAEIQKDVEYRLPFTINN 60
VPS37D ------------------------------------------------------------ 0
VPS37B ------------------------------------------------------------ 0
VPS37C ------------------------------------------------------------ 0

VPS37A LTININILLPPQFPQEKPVISVYPPIRHHLMDKQGVYVTSPLVNNFTMHSDLGKIIQSLL 120
VPS37D ------------------------------------------------------------ 0
VPS37B ------------------------------------------------------------ 0
VPS37C ------------------------------------------------------------ 0

VPS37A DEFWKNPPVLAPTSTAFPYLYSNPSGMSPYASQGFPFLPPYPPQEANRSITSLSVADTVS 180
VPS37D ------------------------------------------------------------ 0
VPS37B ------------------------------------------------------------ 0
VPS37C ------------------------------------------------------------ 0

VPS37A SSTTSHTTAKPAAPSFGVLSNLPLPIPTVDASIPTSQNGFGYKMPDVPDAFPELSELSVS 240
VPS37D ---------------------------MYRA--------RAARAGPEPGSPGRFGILSTG 25
VPS37B --------------------------------------------MAGAGSEARFAGLSLV 16
VPS37C --------------------------------------------------METLKDKTLQ 10
                                                             :   :

VPS37A QLTDMNEQEEVLLEQFLTLPQLKQIITDKDDLVKSIEELARKNLLLEPSLEAKRQTVLDK 300
VPS37D QLRDLLQDEPKLDRIVRLSRKFQGLQLEREACLASNYALAKENLALRPRLEMGRAALAIK 85
VPS37B QLNELLEDEGQLTEMVQKMEETQNVQLNKEMTLASNRSLAEGNLLYQPQLDTLKARLTQK 76
VPS37C ELEELQNDSEAIDQLALESPEVQDLQLEREMALATNRSLAERNLEFQGPLEISRSNLSDR 70
       :* :: ::.  : .      : : :  :::  : :   **. **  .  *:  :  :  :

VPS37A YELLTQMKSTFEKKMQRQHELSESCSASALQARLKVAAHEAEEESDNIAEDFLEGKMEID 360
VPS37D YQELREVAENCADKLQRLEESMHRWSPHCALGWLQAELEEAEQEAEEQMEQLLLGEQSLE 145
VPS37B YQELQVLFEAYQIKKTKLDRQSSSASLETLLALLQAEGAKIEEDTENMAEKFLDGELPLD 136
VPS37C YQELRKLVERCQEQKAKLEKFSSALQPGTLLDLLQVEGMKIEEESEAMAEKFLEGEVPLE 130
       *: *  : .    :  : ..     .       *:.   : *::::   *.:* *:  ::

VPS37A DFLSSFMEKRTICHCRRAKEEKLQQAIAMHSQFHAPL----------------------- 397
VPS37D AFLPAFQRGRALAHLRRTQAEKLQELLRRRERSAQPAPTSAADPPKSFPAAAVLPTGAAR 205
VPS37B SFIDVYQSKRKLAHMRRVKIEKLQEMVLKGQRLPQALAPL--PPRL------------PE 182
VPS37C TFLENFSSMRMLSHLRRVRVEKLQEVVRKPRASQELAGDA--PPPRPPPPVRPVPQGTPP 188
        *:  :   * :.* **.: ****: :

VPS37A ------------------------------------------------------------ 397
VPS37D G---PPA-----------------------VPRSLP------------------------ 215
VPS37B LA---PT---APLPYPAPEASGPP----AVAPRRIPPPPPPVP------AGRLATPFTAA 226
VPS37C VVEEQPQPPLAMPPYPLPYSPSPSLPVGPTAHGALPPAPFPVVSQPSFYSGPLGPTYPAA 248

VPS37A ------------------------------------------------------------ 397
VPS37D -----------PLDSRPVPPLKGSPGCPLGPAPLL------------------SPRPS-- 244
VPS37B M-SSGQAVPYPGLQCPPLPPRVGLP------------------TQQGFSSQFVSPYPP-- 265
VPS37C QLGPRGAAGYSWSPQRSMPPRPGYPGTPMGASGPGYPLRGGRAPSPGYPQQ--SPYPATG 306

VPS37A ---------------------------------------------------- 397
VPS37D ----------------QPEPPHR----------------------------- 251
VPS37B ------------PLPQRPPP--------------------RLPPHQPGFILQ 285
VPS37C GKPPYPIQPQLPSFPGQPQPSVPLQPPYPPGPAPPYGFPPPPGPAWPGY--- 355

CLUSTAL O(1.2.4) multiple sequence alignment
```

c

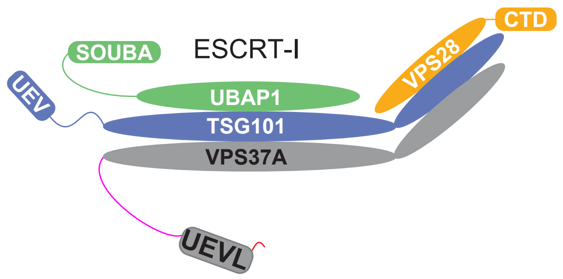

Supplementary Fig. 1: VPS37A predicted structure, sequence alignment, and ESCRT-I schematic representation.

a Domain structure of VPS37A (top) and its AlphaFold predicted structure (bottom). The N-terminal 148 residues containing the UEVL domain is shown in green, while the C-terminal 180 residues containing a modifier of the rudimentary[Mod(r)] domain is shown in cyan. A long disordered stretch between the N- and C-terminal domains is shown in magenta. The AlphaFold structure was obtained from: http://www.alphafold.ebi.ac.uk/entry/Q8NEZ2.

b Sequence alignment of human VPS37 homologs by CLUSTAL O(1.2.4).
UniProt ID: Q8NEZ2 for VPS37A, Q86XT2 for VPS37D, Q9H9H4 for VPS37B, and A5D8V6 for VPS37C.
The VPS37A has a unique N-terminal domain.

c Schematic representation of the ESCRT-I complex constructed based on previous reports: (Wunderley et al. J. Cell. Sci. (2014), 127 (3): 663–672; Agromayor et al. Structure (2012), 20: 414-428.)

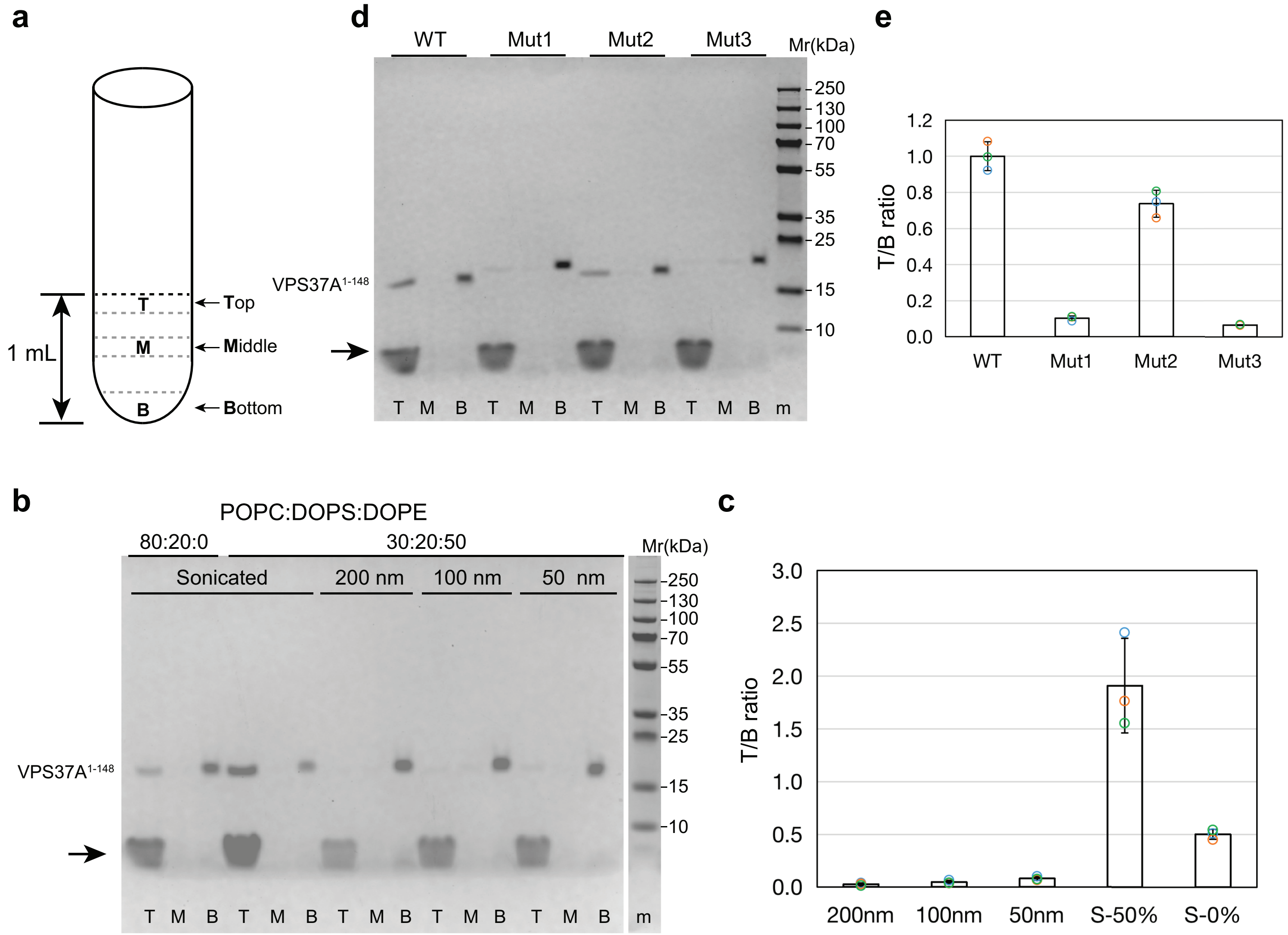

Supplementary Fig. 2: VPS37A$^{1-148}$ selectively interacts with highly curved membranes in a flotation assay using liposomes with negatively charged DOPS.

a Schematic diagram of the sample in the flotation experiment. T, M, and B represent top, middle, and bottom layers, respectively, after centrifugation.

b Gel images of liposome flotation assays for VPS37A$^{1-148}$ mixed with sonicated or extruded liposomes with membrane pore sizes of 50, 100, or 200 nm (POPC:DOPS:DOPE=30:20:50, or 80:20:0, 800 μM; protein:lipid=1:400, molar ratio). T, M, and B represent top, middle, and bottom layers after centrifugation. Protein marker is indicated by m. Arrow indicates the lipid band. Amount of VPS37A$^{1-148}$ in the top layer relative to that in the bottom layer (T/B) is quantitated by ImageJ and plotted in (**c**).

c Sonicated liposomes containing 0 or 50% PE (molar ratio), referred to as S-0% or S-50%, respectively. Data are presented as mean ± SD (standard deviation). Quantifications are obtained from three separate measurements (n=3).

d Gel images of liposome flotation assays for VPS37A$^{1-148}$ wildtype (WT), Mut1, Mut2, and Mut3 using sonicated liposomes (POPC:DOPS:DOPE=30:20:50, 800 μM; protein:lipid=1:400, molar ratio). T, M, and B represent top, middle, and bottom layers after centrifugation. Protein marker is indicated by m. Arrow indicates the lipid band. Amount of protein in the top layer relative to that in the bottom (T/B) is quantitated by ImageJ and plotted in (**e**).

e The T/B ratio of mutants is normalized to the ratio of of VPS37A$^{1-148}$ WT using the same batch of sonicated liposomes. Data are presented as mean ± SD from three independent experiments (n=3 for each construct).

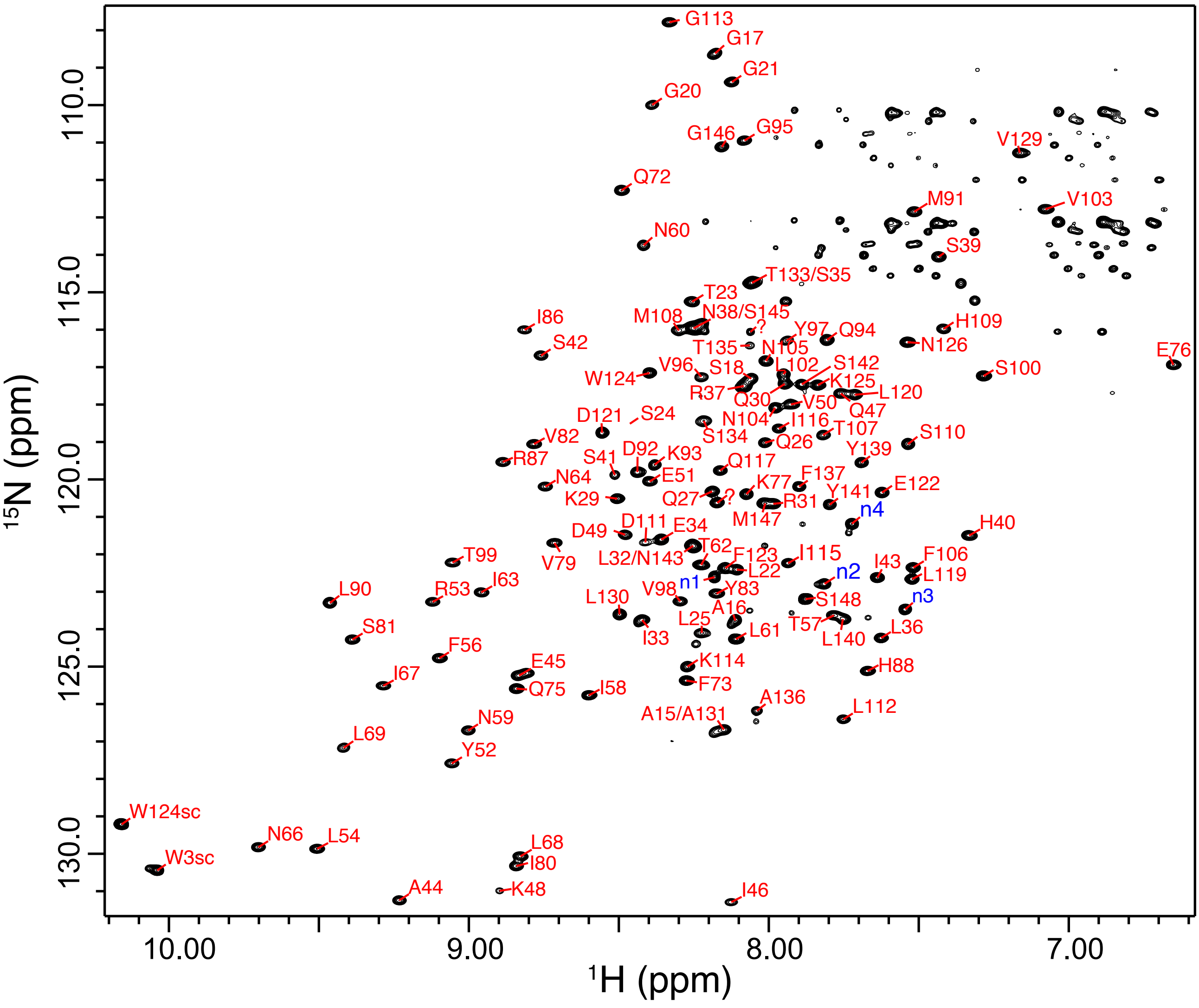

Supplementary Fig. 3: ^{1}H-^{15}N TROSY spectrum acquired on a Bruker 600 MHz spectrometer at 25 °C, pH 6.5 for ^{15}N, ^{13}C-labeled VPS37A$^{1\text{-}148}$ with resonance assignments. The unassigned residues from the N-terminus (n1 to n4) are indicated.

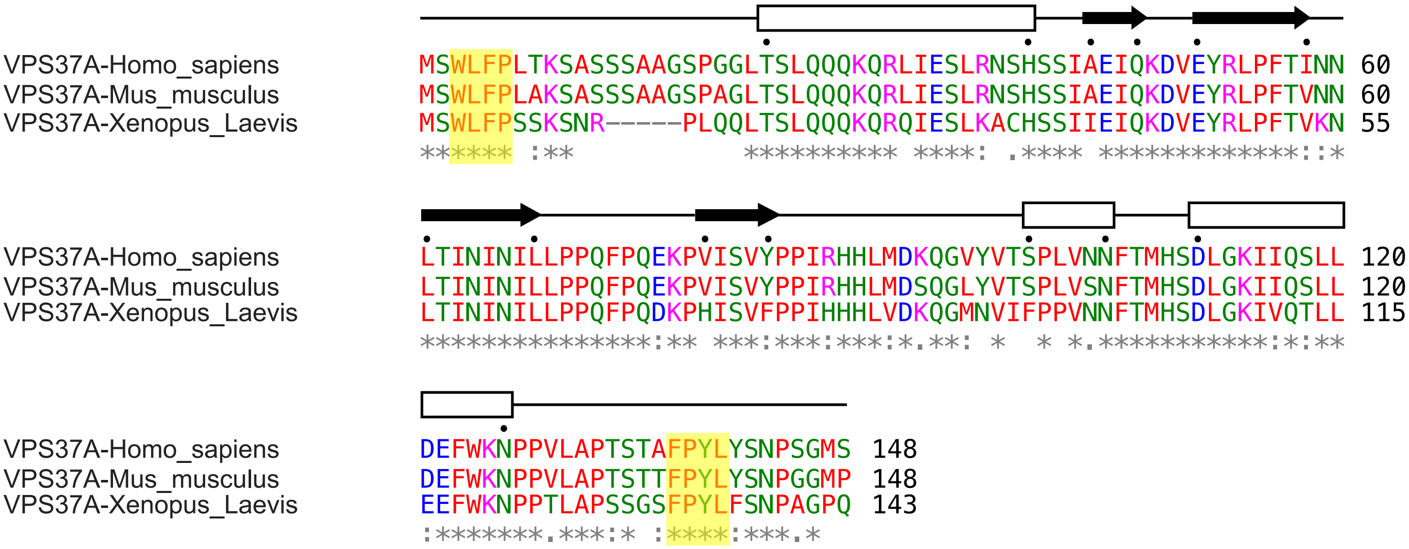

Supplementary Fig. 4: Sequence alignment of VPS37A N-terminal domain from different species.

Two highly conserved regions with bulky hydrophobic amino acids which precede and follow the UEVL domain are highlighted in yellow. The secondary structure is indicated above the sequence. A single line represents a disordered region or loop, a rectangle represents an α-helix, and an arrow represents β-sheet. The dot indicates the start or end of a secondary structure.

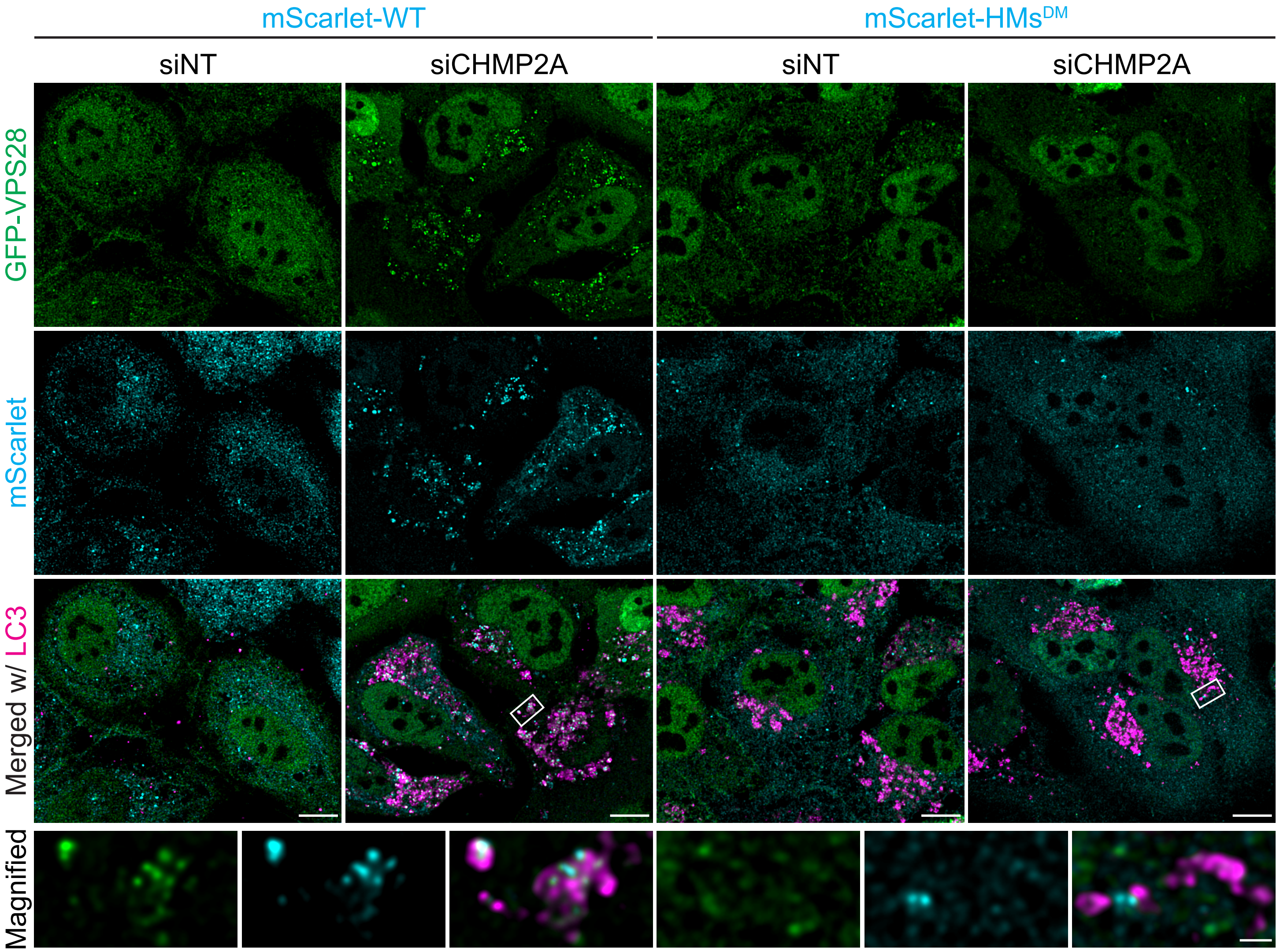

Supplementary Fig. 5: Hydrophobic membrane-binding motifs in the VPS37A N-terminal domain mediate ESCRT-I targeting to the phagophore.

Confocal images of VPS37A KO cells that were stably transduced with GFP-VPS28 and mScarlet-VPS37A WT or HMs^{DM}, and transfected with the indicated siRNAs for 45 hrs and starved for 3 hrs. Magnified images in the boxed areas are shown in the bottom panels. Scale bars represent 10 μm, and 1 μm in the magnified images.

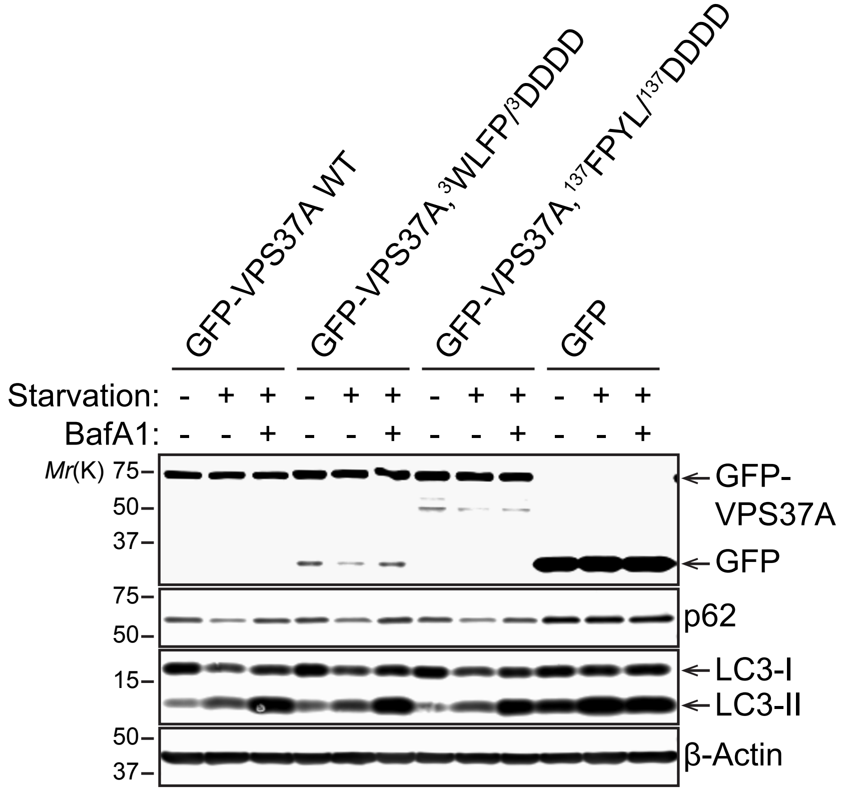

Supplementary Fig. 6: Two hydrophobic membrane binding motifs in the VPS37A N-terminal domain are functionally redundant in autophagy.

Immunoblot analysis of VPS37A KO cells that were stably transduced with GFP-VPS37A WT, ^{3}WLFP/^{3}DDDD mutant, ^{137}FPYL/^{137}DDDD mutant, or control GFP and starved for 3 hrs in the presence or absence of BafA1.

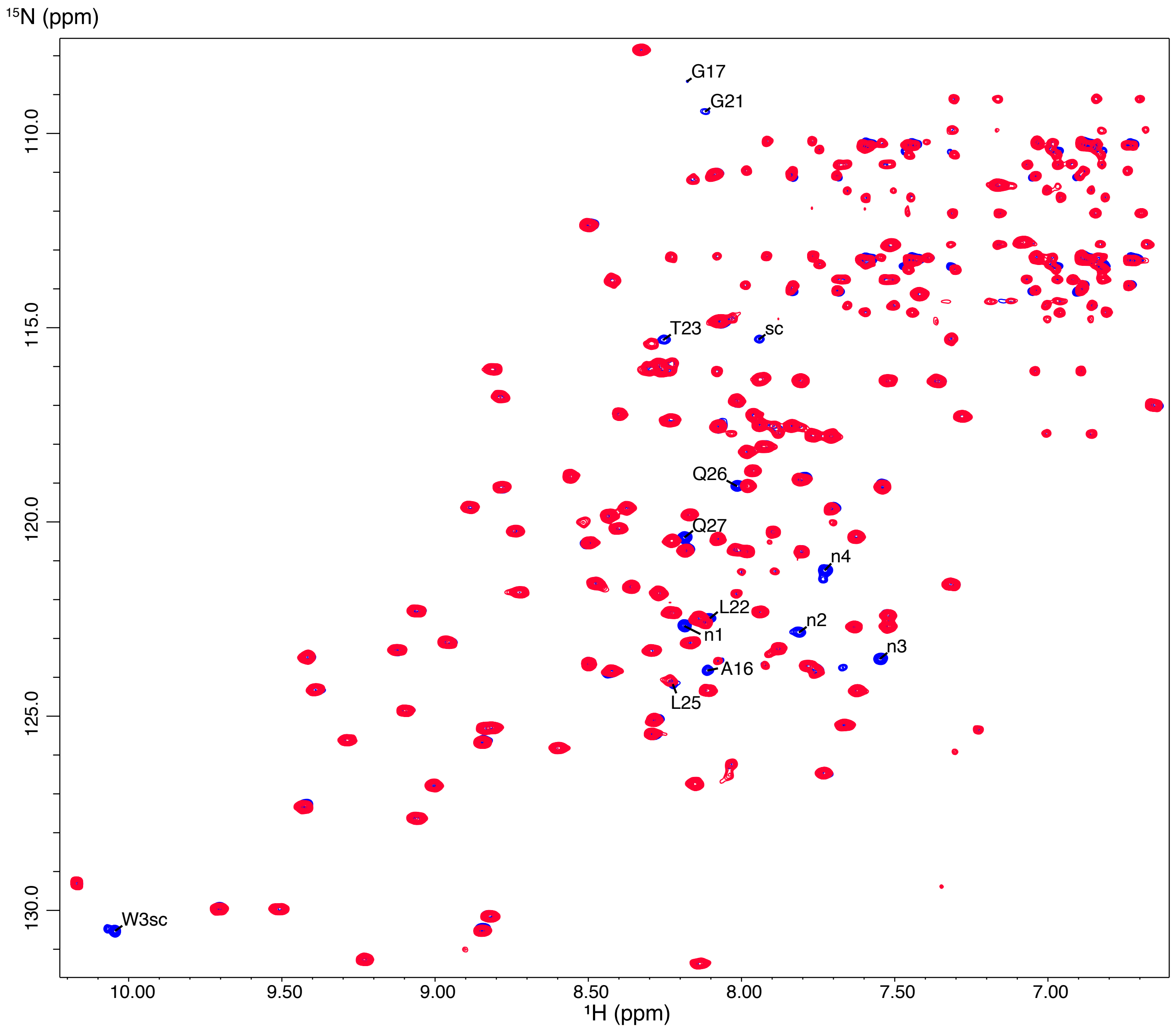

Supplementary Fig. 7: Deletion of first 20 residues of N-terminal domain does not perturb VPS37A UEVL structure.

Overlay of ^{15}N-labeled VPS37A$^{1\text{-}148}$ (blue) and VPS37A$^{21\text{-}148}$ (red) TROSY spectra acquired on a Bruker 600 MHz spectrometer at 25 °C. Perturbed, unassigned (from the N-terminus), and sidechain resonances are indicated with their assignments, n1 to n4, and sc, respectively.

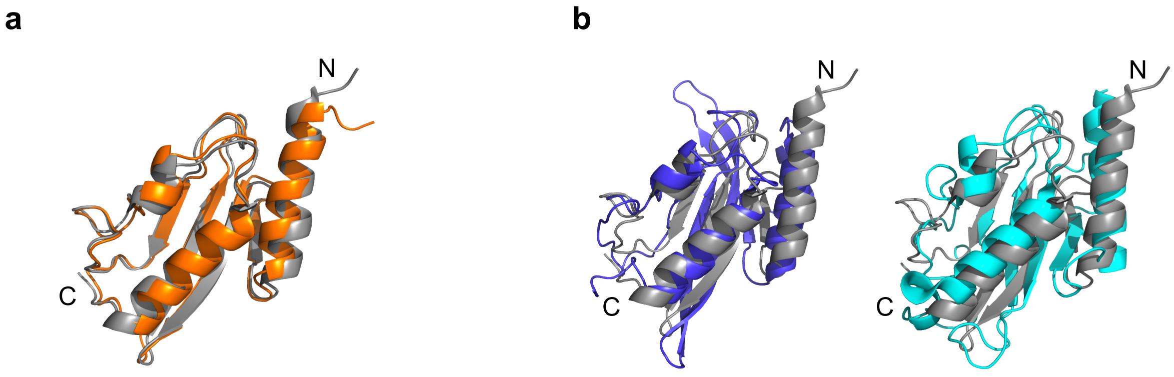

Supplementary Fig. 8: Structural comparisons for VPS37A$^{21\text{-}148}$.

a Structural alignments of VPS37A$^{21\text{-}148}$. Gray, NMR structure (PDB 8E22); Orange, AlphaFold predicted structure (https://www.alphafold.ebi.ac.uk/entry/Q8NEZ2).

b Structural alignments of VPS37A$^{21\text{-}148}$ (gray, PDB 8E22) with: left, TSG101 UEV structure (tv-blue, PDB 1KPQ); right, human Mms2 structure (cyan, PDB 1ZGU).

The C-terminal disordered region (residues 132 to 148) of VPS37A is not shown for clarity.

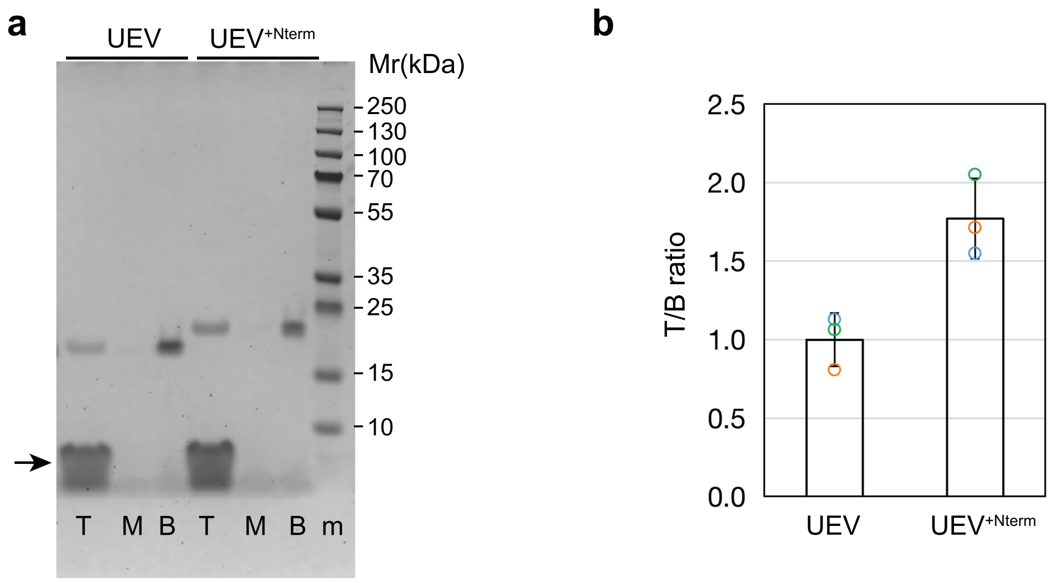

Supplementary Fig. 9: The [3]WLFP motif of VPS37A enhances membrane binding of TSG101 UEV.

a Gel images of liposome flotation assays for TSG101 UEV and a mutant of TSG100 UEV with the addition of VPS37A N-terminal first 20 residues (TSG101 UEV^{+Nterm}) using 800 μM sonicated liposomes (POPC:DOPG:DOPE=30:20:50, protein:lipid=1:400, molar ratio) in 50 mM HEPES, 150 mM NaCl, pH 7.5, and 1 mM TCEP. T, M, and B represent top, middle, and bottom layers respectively after density gradient ultracentrifugation. The marker is indicated by m. Arrow indicates the lipid band. Amount of protein in the top layer relative to that in the bottom (T/B) is quantitated by ImageJ and plotted in (**b**).

b Plots of TSG101 UEV and UEV^{+Nterm} in the top versus bottom layers for sonicated liposomes. (POPC:DOPG:DOPE=30:20:50, protein:lipid=1:400, molar ratio). The T/B ratio of UEV^{+Nterm} is normalized to the ratio of TSG101 UEV using the same batch of sonicated liposomes. Amount of proteins in the top layer relative to the bottom layer is quantitated by ImageJ. Data are presented as mean ± SD from three independent experiments (n=3 for each construct)

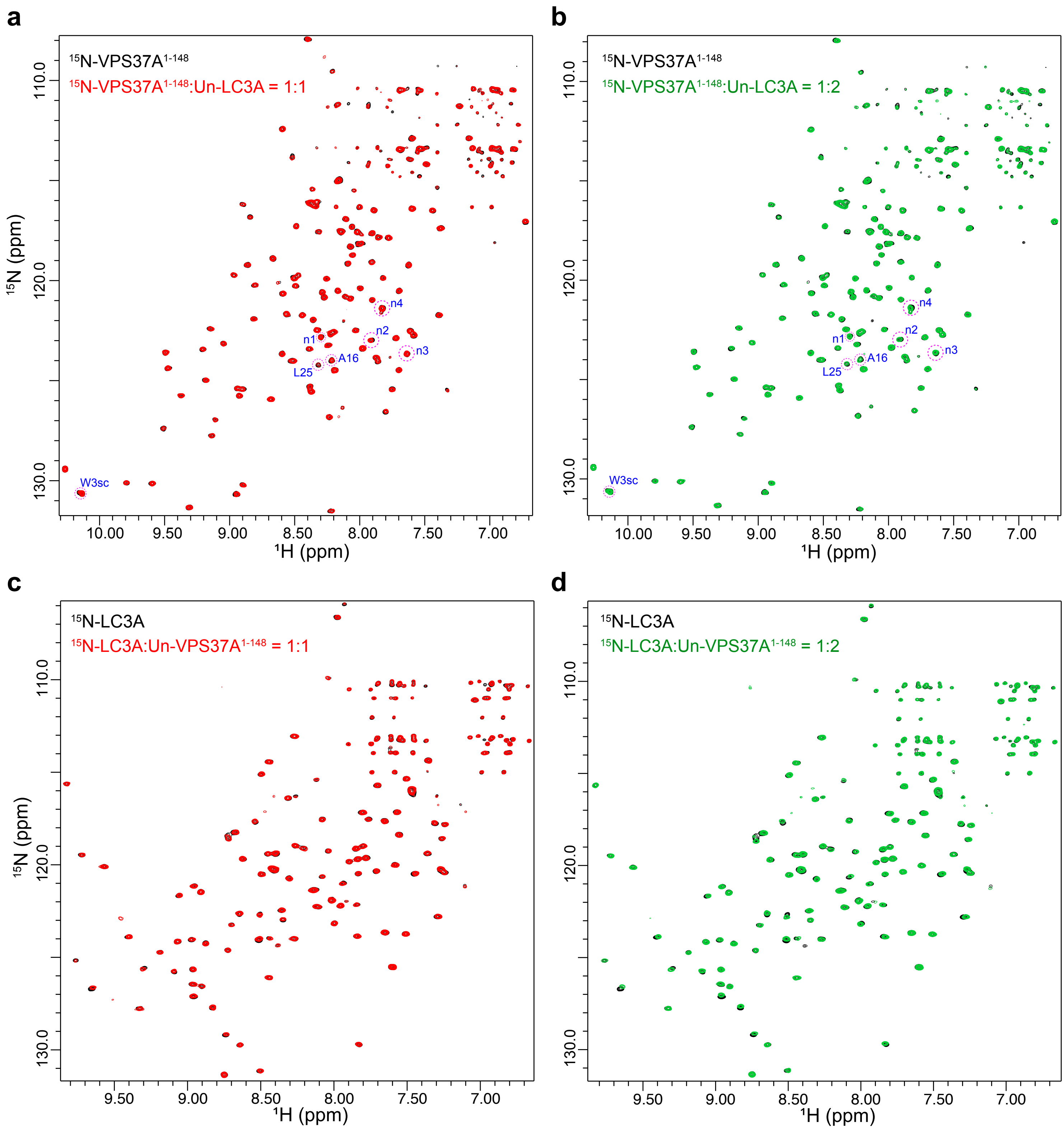

Supplementary Fig. 10: VPS37A$^{1-148}$ does not interact with human LC3A.

a, b Overlay of 2D ^{1}H-^{15}N correlation spectra of ^{15}N-labeled VPS37A$^{1-148}$ (50 μM) without (black) and with unlabeled-LC3A (Un-LC3A, 50 μM, red, **a**; 100 μM, green, **b**) in 50 mM HEPES, 150 mM NaCl, pH 7.0, and 1 mM TCEP. Several N-terminal resonances of VPS37A$^{1-148}$ are indicated.

c, d Overlay of 2D ^{1}H-^{15}N correlation spectra of ^{15}N-labeled LC3A (50 μM) without (black) and with unlabeled-VPS37A$^{1-148}$ (Un-VPS37A$^{1-148}$, 50 μM, red, **c**; 100 μM, green, **d**) in 50 mM HEPES, 150 mM NaCl, pH 7.0, and 1 mM TCEP.

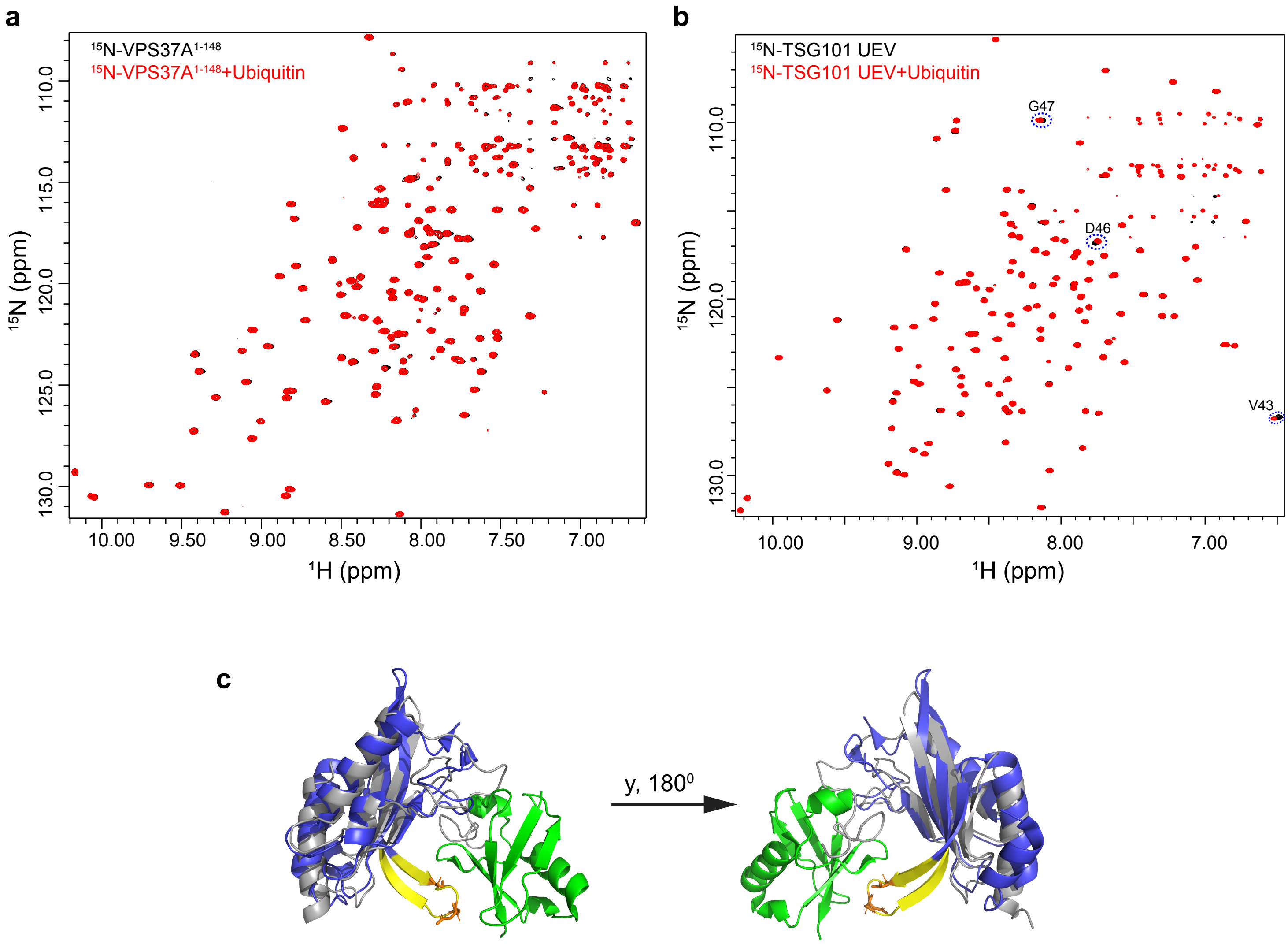

Supplementary Fig. 11: VPS37A$^{1\text{-}148}$ domain does not interact with Ubiquitin.

a, Overlay of 2D ^{1}H-^{15}N correlation spectra of ^{15}N-labeled VPS37A$^{1\text{-}148}$ (50 μM) without (black) and with (red) Ubiquitin (100 μM) in a buffer of 50 mM HEPES, 150 mM NaCl, pH 6.8, and 2 mM TCEP.

b, Overlay of 2D ^{1}H-^{15}N correlation spectra of ^{15}N-labeled TSG101 UEV (50 μM) without (black) and with (red) Ubiquitin (100 μM) in a buffer of 50 mM HEPES, 150 mM NaCl, pH 6.8, and 2 mM TCEP. The perturbed resonances are labeled with their assignments (BMRB accession code: 50765).

c, Structural alignment of VPS37A$^{21\text{-}148}$ (gray, PDB 8E22) with Ubiquitin-bound TSG101 UEV (blue, PDB 1S1Q). The β-tongue of TSG101 UEV is shown in yellow and residues perturbed by the binding of Ubiquitin (green) are shown in orange sticks. The Ubiquitin-binding region in the TSG101 UEV (yellow region) is absent in the VPS37A UEVL.

Ubiquitin was purchased from R&D Systems (U-100H-10M).

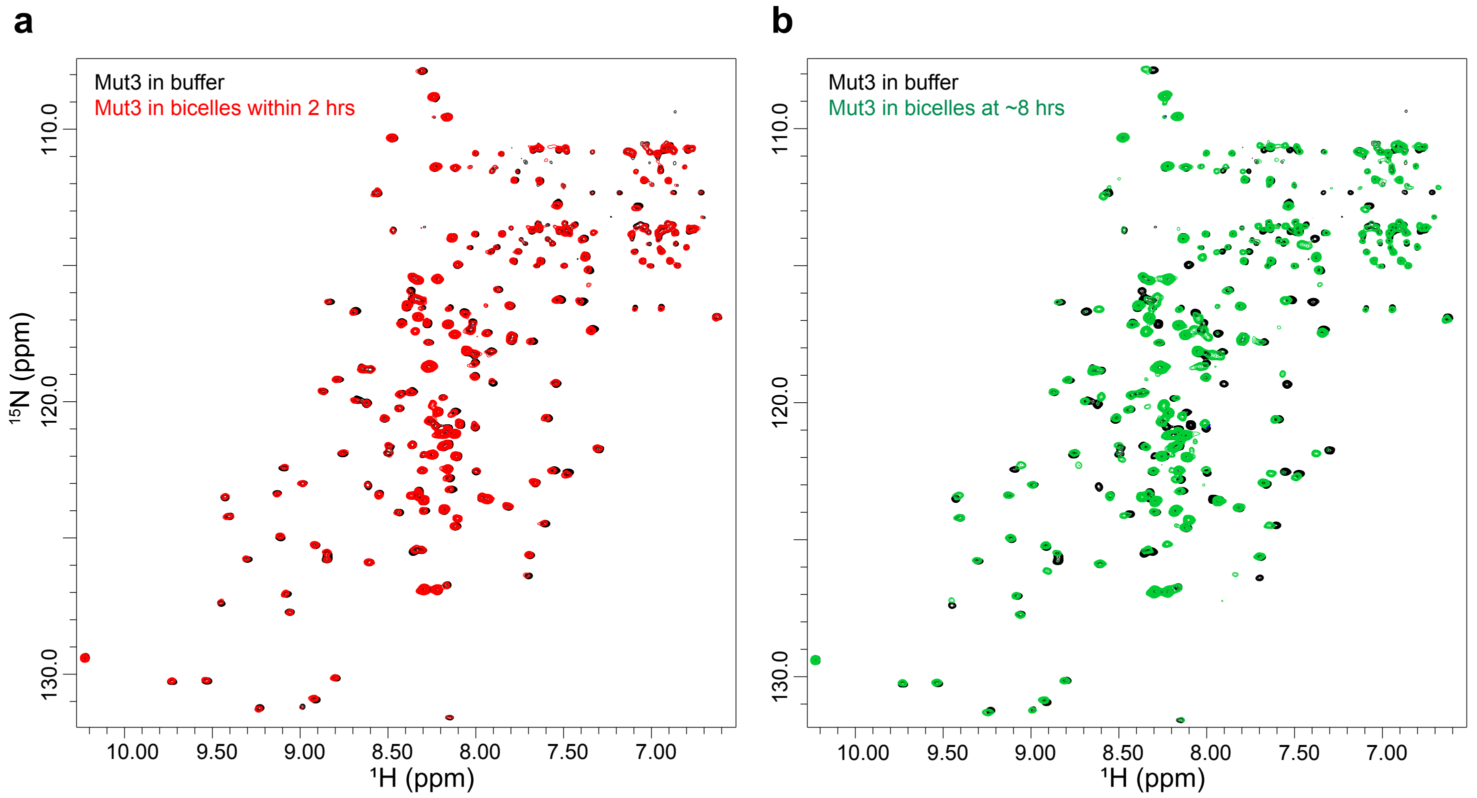

Supplementary Fig. 12: VPS37A UEVL domain/bicelle interactions.

Overlay of 2D ^{1}H-^{15}N correlation spectra of a ^{15}N-labeled VPS37A$^{1\text{-}148}$ variant with ^{3}WLFP/^{3}DDDD and ^{137}FPYL/^{137}DDDD mutations (Mut3) without (black, **a** and **b**) and with bicelles (DMPC:DMPG:DHPC = 8:2:20, molar ratio) within 2 hrs (red, **a**) or at ~8 hrs (green, **b**) in 25 mM HEPES, 150 mM NaCl, pH 7.0. NMR spectra were collected at 288K.

Fig. 3a

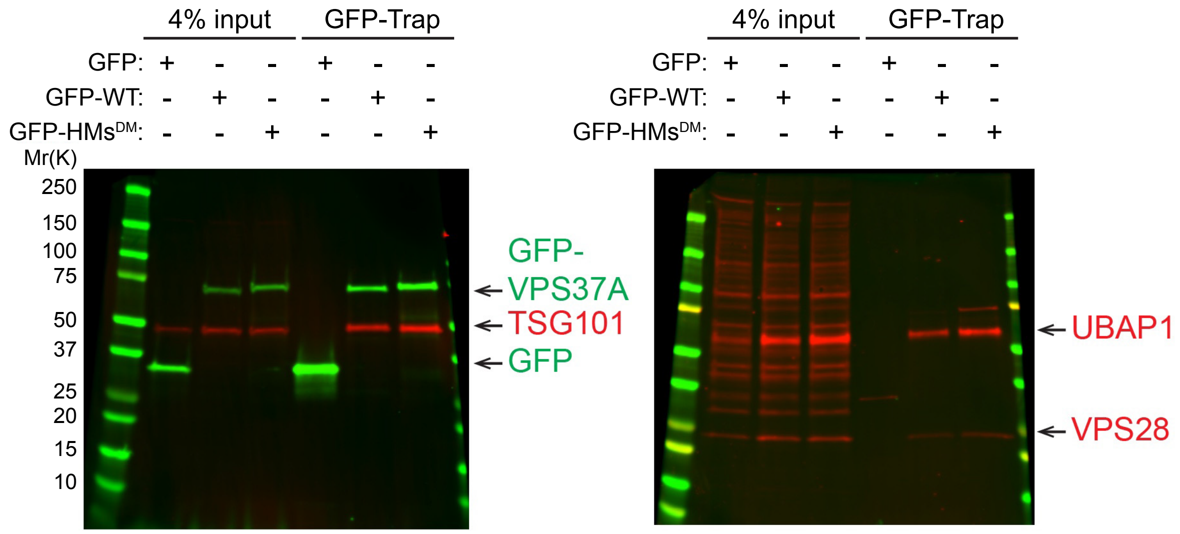

Fig. 3f

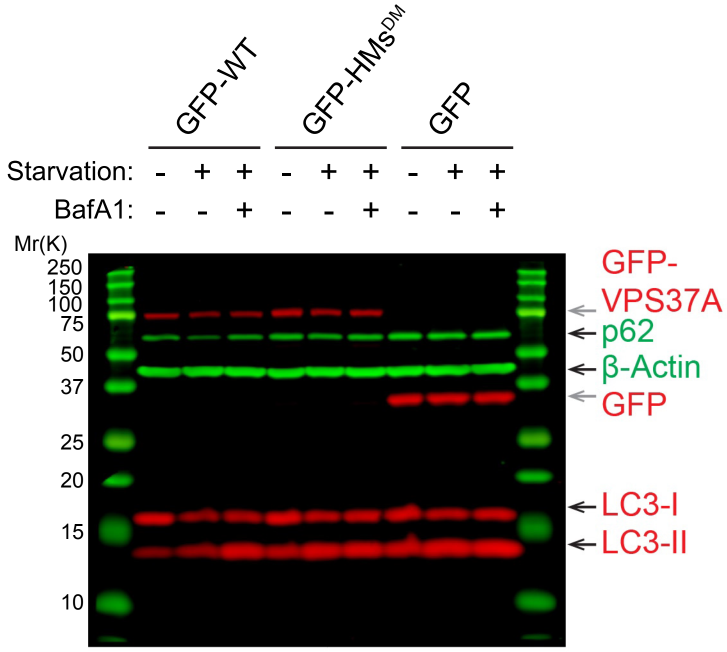

Supplementary Fig. 13: Original immunoblots for Fig. 3a, f.

Fig. 4c

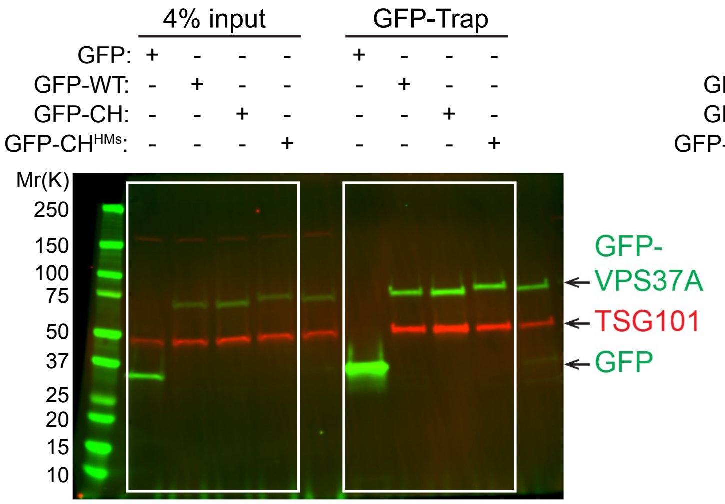

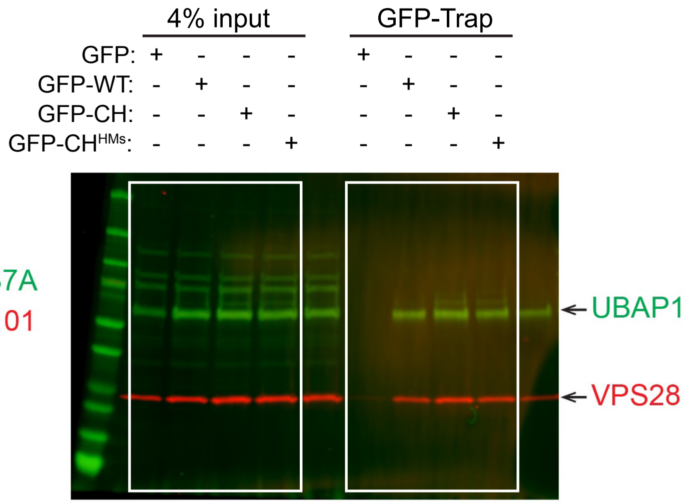

Fig. 4h

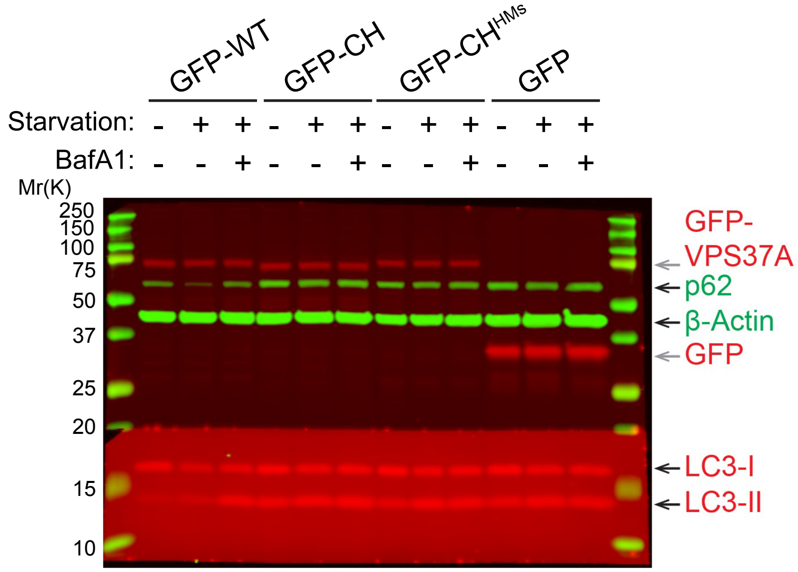

Supplementary Fig. 14: Original immunoblots for Fig. 4c, h.

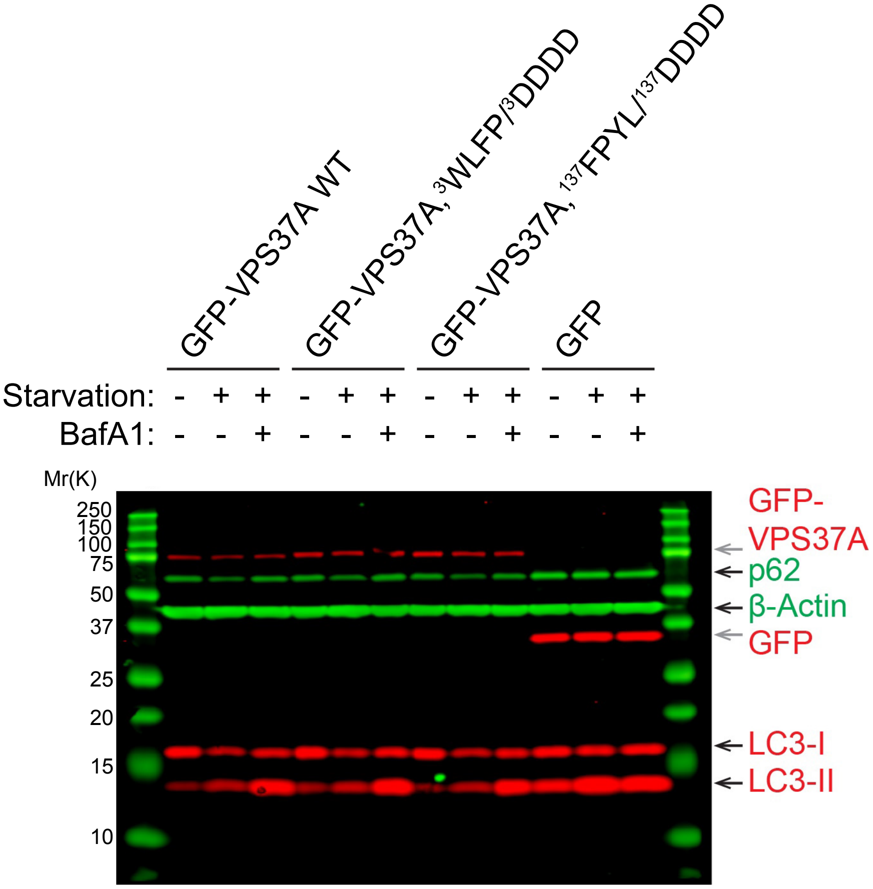

Supplementary Fig. 15: Original immunoblots for Supplementary Fig. 6.

Supplementary Table 1 | Primers (F, Forward; R, Reverse) used in this study.

**Primers ID for *in vitro* experiments**	**Sequences from 5' to 3'**
VPS37A$^{1-148}$_F	AGTGCCTCGCGGATCCATGAGCTGGCTTTTTC
VPS37A$^{1-148}$_R	GGTGGTGGTGCTCGAGCTAAGACATCCCACTT
VPS37A$^{21-148}$_F	GGCCTCACCAGCCTCCAG
VPS37A$^{21-148}$_R	GGATCCGCGAGGCACTAG
Mut1_F	GATGACCTGACCAAGAGCGCCTC
Mut1_R	ATCGTCGCTCATGGATCCGCGAGG
Mut2_F	GATGACTACAGTAACCCAAGTGGG
Mut2_R	ATCATCTGCTGTTGAAGTAGGAGC
Mut4_F	CGGTGGCGGTGGCCTCACCAGCCTC
Mut4_R	CCACCGCCGGGAAAAAGCCAGCTCATGGATC
Mut5_F	TTTTCCCGGCGGTGGCGGTGGTGGCCTCACC AGCCTC
Mut5_R	AGCCAGCCACCGCCACCGCCGGGAAAAAGCC AGCTCATGGATC
Mut6_F	ATCTAGGCGGTGGCGGTGGCGGT
Mut6_R	AAGGAAAGCTCATGGATCCGCGAGGCACTAGC
Mut7_F	GAGCTGGCTTCCCTTTGGCGGTGGCG
Mut7_R	ATGGATCCGCGAGGCACT
Mut8_F	TTTTGGCGGTGGCGGTGGCGGT
Mut8_R	AGGGGCCAGCTCATGGATCCGCGAGGC
TSG101 UEV_F1	AGTGCCTCGCGGATCCATGGCGGTGTCGGAG AGC
TSG101 UEV_F2	GGTGGTGGTGCTCGAGCTACGGTCGTGAAAAT ACAGGTGG
TSG101 UEV^{+Nterm}_F	GCCTCCTCCTCCGCGGCTGGGTCCCCCGGTA TGGCGGTGTCGGAGAGC
TSG101 UEV^{+Nterm}_R	GCTCTTGGTCAGGGGAAAAAGCCAGCTCATGG ATCCGCGAGGCACTAG
**Primers ID for in cell experiments**	**Sequences from 5' to 3'**
pCDH1-GFP-HM1_Mut-Puro, pCDH1-GFP-HM_DM-Puro_F	TACAAGTCCGGACTCAGATCTCGAGCTCAAAG CGACGACGACGACCTGACCAAGAGCGCCTCC
pCDH1-GFP-HM1_Mut-Puro, pCDH1-GFP-HM_DM-Puro_R	CTGAGAGTTCTGGAAATGCATCAGGGACATCT GGCATC

pCDH1-GFP-HM2_Mut-Puro, pCDH1-GFP-HM_DM-Puro_F1	TACAAGTCCGGACTCAGATCTCGAGCTCAAAGCTGGCTTTTTC
pCDH1-GFP-HM2_Mut-Puro, pCDH1-GFP-HM_DM-Puro_R1	GGTTACTGTAGTCGTCATCGTCTGCTGTTGAAGTAGGAGCTAAAAC
pCDH1-GFP-HM2_Mut-Puro, pCDH1-GFP-HM_DM-Puro_F2	GACGATGACGACTACAGTAACCCAAGTGGG
pCDH1-GFP-HM2_Mut-Puro, pCDH1-GFP-HM_DM-Puro_R2	CTGAGAGTTCTGGAAATGCATCAGGGACATCTGGCATC
pCDH1-CMV-GFP-CH-Puro_F1	TACAAGTCCGGACTCAGATCTCGAGCTCAAGCTTCGAATTC
pCDH1-CMV-GFP-CH-Puro_R1	CGGTCGTGAAAATACAGG
pCDH1-CMV-GFP-CH-Puro_F2	CACCTGTATTTTCACGACCGTATGCTTCTCAGGGTTTTC
pCDH1-CMV-GFP-CH-Puro_R2	CGCAGATCCTTGCGGCCGCGGATCCCTATAGTGGAGCATG
pCDH1-CMV-GFP-CH_HMs-Puro_F1	TACAAGTCCGGACTCAGATCTCGAGCTCAAAGCTGGCTTTTTCCCCTGAC
pCDH1-CMV-GFP-CH_HMs-Puro_R1	CCGACACCGCACCGGGGGACCCAGCCGC
pCDH1-CMV-GFP-CH_HMs-Puro_F2	GTCCCCCGGTGCGGTGTCGGAGAGCCAG
pCDH1-CMV-GFP-CH_HMs-Puro_R2	CTGTTGAAGTCGGTCGTGAAAATACAGGTGGC
pCDH1-CMV-GFP-CH_HMs-Puro_F3	TTCACGACCGACTTCAACAGCATTTCCTTATC
pCDH1-CMV-GFP-CH_HMs-Puro_R3	CGCAGATCCTTGCGGCCGCGGATCCCTATAGTGGAGCATG
