# Supplementary material for: Identification of membrane curvature sensing motifs essential for VPS37A phagophore recruitment and autophagosome closure
Source: Commun Biol. 2024 Mar 15;7:334. doi: 10.1038/s42003-024-06026-7 (PMC10942982; doi:10.1038/s42003-024-06026-7)
Supplement: Supplementary file 2 — Description of Additional Supplementary Files [file 42003_2024_6026_MOESM2_ESM.pdf]

Description of Additional Supplementary Files

File name: Supplementary data 1

Description: Source data underlying graphs and display items in Figures and SI file
